# Simultaneous spatial transcriptomics and morphology profiling as tools to explore how microglia change with age

**DOI:** 10.1038/s43587-026-01089-z

**Published:** 2026-03-10

**Authors:** Douglas E. Henze, Andy P. Tsai, Tony Wyss-Coray, Stephen R. Quake

**Affiliations:** 1 Department of Bioengineering, Stanford University, Stanford, CA, USA.; 2 Department of Neurology and Neurological Sciences, Stanford University School of Medicine, Stanford, CA, USA.; 3 Wu Tsai Neurosciences Institute, Stanford University, Stanford, CA, USA.; 4 The Phil and Penny Knight Initiative for Brain Resilience, Stanford University, Stanford, CA, USA.; 5 Department of Applied Physics, Stanford University, Stanford, CA, USA.

## Abstract

Cellular morphology is tightly linked to function, but how subcellular transcript localization contributes remains unclear. Using microglia, the brain’s resident macrophages, as a model, we combined multiplexed error-robust fluorescence in situ hybridization with immunohistochemistry to map how morphology and subcellular mRNA localization interact with function in young and aged mouse brains. We show that mRNA spatial organization varies across microglial states and defines distinct localization patterns within their processes, revealing morphological heterogeneity within transcriptomically defined populations. Notably, we found a subpopulation of disease-associated-like microglia with a ramified morphology (that is, displaying numerous processes), challenging the conventional assumption between morphology and microglial states. Finally, we found that aging may reshape mRNA distributions and their co-localization networks, shifting microglial programs from intracellular signaling and regulation of phagocytosis toward migration and catabolic regulation. Our findings highlight the role of subcellular transcript organization in shaping microglial morphology and function, offering new avenues for studying and modulating microglial states in health, disease and aging.

Subcellular localization of mRNA provides precise spatial control over protein synthesis^1^. This process is essential for cells with complex morphologies, and its disruption has been linked to age-related neurodegenerative diseases, including Alzheimer’s disease^2–8^. In the central nervous system (CNS), neurons and glia rely on localized transcripts to maintain polarized functions^9–13^. Microglia, the resident immune cells of the CNS^14^, are an ideal model for studying how mRNA localization’s interaction with cellular morphology generates functional diversity^15,16^. Microglia have traditionally been classified into two morphological states with distinct functions: a ‘homeostatic’ state with ramified or branched processes and an ‘activated’ (disease-associated) state with an amoeboid shape^17^. This variation has long been assumed to reflect the distinct functional specializations of transcriptomically defined states, with the extensive branching of ramified microglia enabling surveillance of large tissue volumes while the compact amoeboid form facilitates efficient phagocytosis of cellular debris^18,19^. However, the mapping between microglial morphology and function may be more complex than this simple picture suggests, as recent findings showed that ramified microglia can perform phagocytic functions typically associated with amoeboid morphology^20^.

Single-cell transcriptomics has characterized myriad microglial states^21–23^, including homeostatic^24^, transitioning^25^ and disease-associated microglia (DAM)^26^, across healthy and diseased brains^27,28^. However, these studies relied on tissue dissociation, which obscures spatial context and prevents measurement of cellular morphology and subcellular transcript localization. By contrast, spatial transcriptomics preserves tissue architecture, but most approaches lack the resolution to resolve subcellular mRNA distributions^29^. Techniques such as multiplexed error-robust fluorescence in situ hybridization (MERFISH) address this limitation, enabling high-resolution mRNA mapping and studies of aging and disease in the CNS^30–32^. Although a recent study combined spatial transcriptomics with electron microscopy on adjacent sections to correlate microglial ultrastructure with the transcriptome^33^, no current method captures subcellular transcript location along the full extent of their processes. Emerging evidence suggests that mRNA positioning within microglial processes contributes to cell function^34^.

To address this, we combined MERFISH with high-content fluorescence immunostaining to examine how transcript localization correlates with microglial morphology in young (3-month-old) and aged (24-month-old) mouse brains. Our analysis uncovered diverse microglial morphologies associated with distinct transcriptomic phenotypes, including a subpopulation with a DAM signature with highly ramified processes. Correlating transcriptomic data with morphological features, we identified genes strongly associated with this cell state, potentially impacting microglial function. Spatial mapping of transcripts within individual microglia revealed that process-localized genes may be predictive of specific morphological states across the lifespan. Aging altered the distribution of compartmentalized mRNAs and reshaped their co-localization networks, shifting microglial programs from cytokine production and phagocytic processes in younger brains toward migration and catabolic pathways in older brains. Collectively, these findings show that subcellular mRNA organization, in both localization and age-dependent clustering, helps define distinct morphological phenotypes and functional states of microglia and illuminates how localized transcriptomic architecture shapes microglial roles in the aging brain.

## Results

### Microglial transcriptional state depends on age and region

**Extended Data Fig. 1 |:**
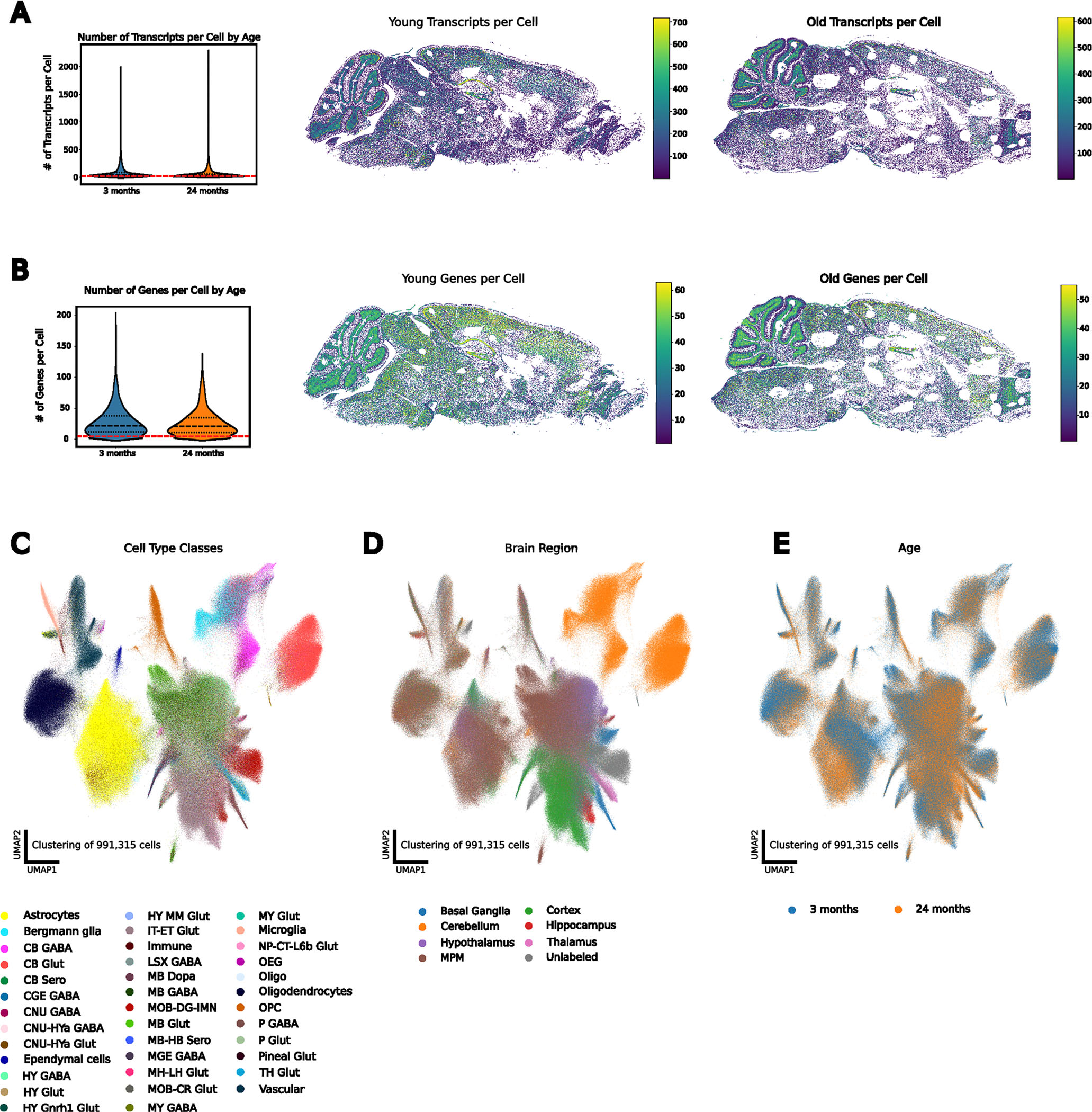
Spatial Transcriptomics Resolves Cell Types Across the Aging Mouse Brain. **A**, Number of cells passing minimum number of transcripts per cell threshold (red line) (left) with a representative spatial heatmap in a young (middle) and old (right) brain. **B**, Number of cells passing minimum number of genes threshold (red line) (left) with a representative spatial heatmap in a young (middle) and old (right) brain. **C**, UMAP representation of the spatial transcriptome of all cells passing QC (n = 991,315), colored by major cell class. **D**, UMAP representation of all cells passing QC, colored by brain region. **E**, UMAP representation of all cells passing QC, colored by age. The violin plots are demarcated with median values and lines representing the first and third quartiles.

**Fig. 1 |:**
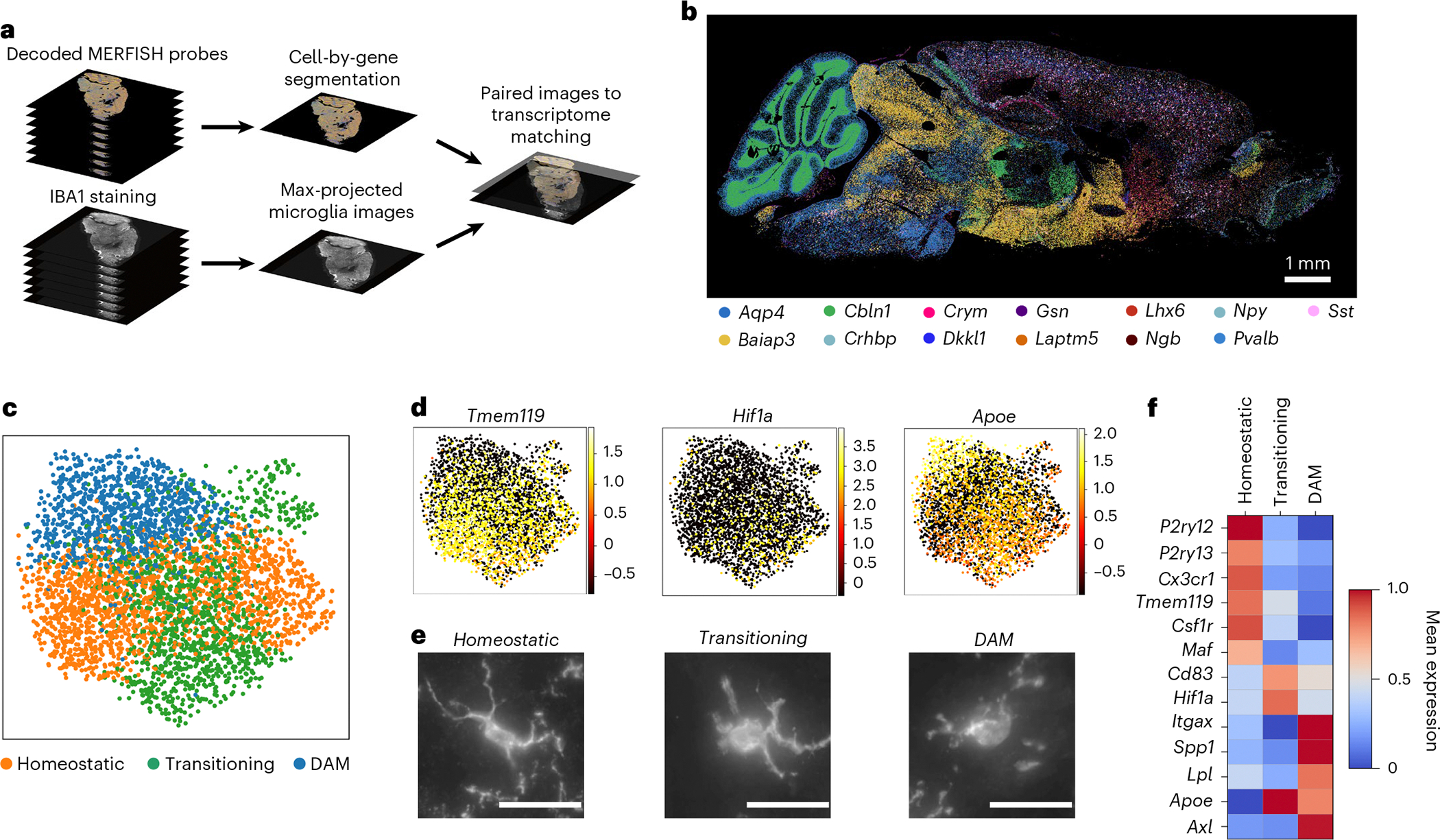
MERFISH paired with fluorescence immunohistchemistry allows for single microglia transcriptomics and imaging. **a**, Schematic overview of the image processing pipeline to generate maximum-projected transcriptome decodings and images of microglia across the whole mouse brain. **b**, Neuronal and glial markers resolved in space. **c**, UMAP plots of microglia colored by identified clusters. **d**, UMAP plots of microglia (*n* = 3,970 cells) colored by marker gene expression. **e**, Representative images of IBA1-stained microglia from each of the identified clusters. Scale bars, 25 μm. **f**, Heatmap of average expression for each microglia cluster.

To visualize gene expression and microglial morphology in situ, we applied subcellular-resolution MERFISH combined with fluorescence immunostaining of ionized calcium-binding adaptor molecule 1 (IBA1)^35^ on sagittal sections of three mouse brains per sex at 3 months old (young) and 24 months old (aged) (12 in total) (Fig. 1a and Methods). Our spatial transcriptomics dataset contains counts and subcellular localization for a panel of 500 curated genes, designed to capture cell type and age-related variation across the brain (Fig. 1b and Methods). This gene panel was used to segment^36^ and characterize spatially resolved single-cell gene expression. After quality control (Extended Data Fig. 1a,b), preprocessing and clustering of spatially resolved single-cell transcriptomes, we identified and annotated^30^ 991,315 cells across 38 major cell classes spanning seven brain regions (Methods and Extended Data Fig. 1c–e).

**Extended Data Fig. 2 |:**
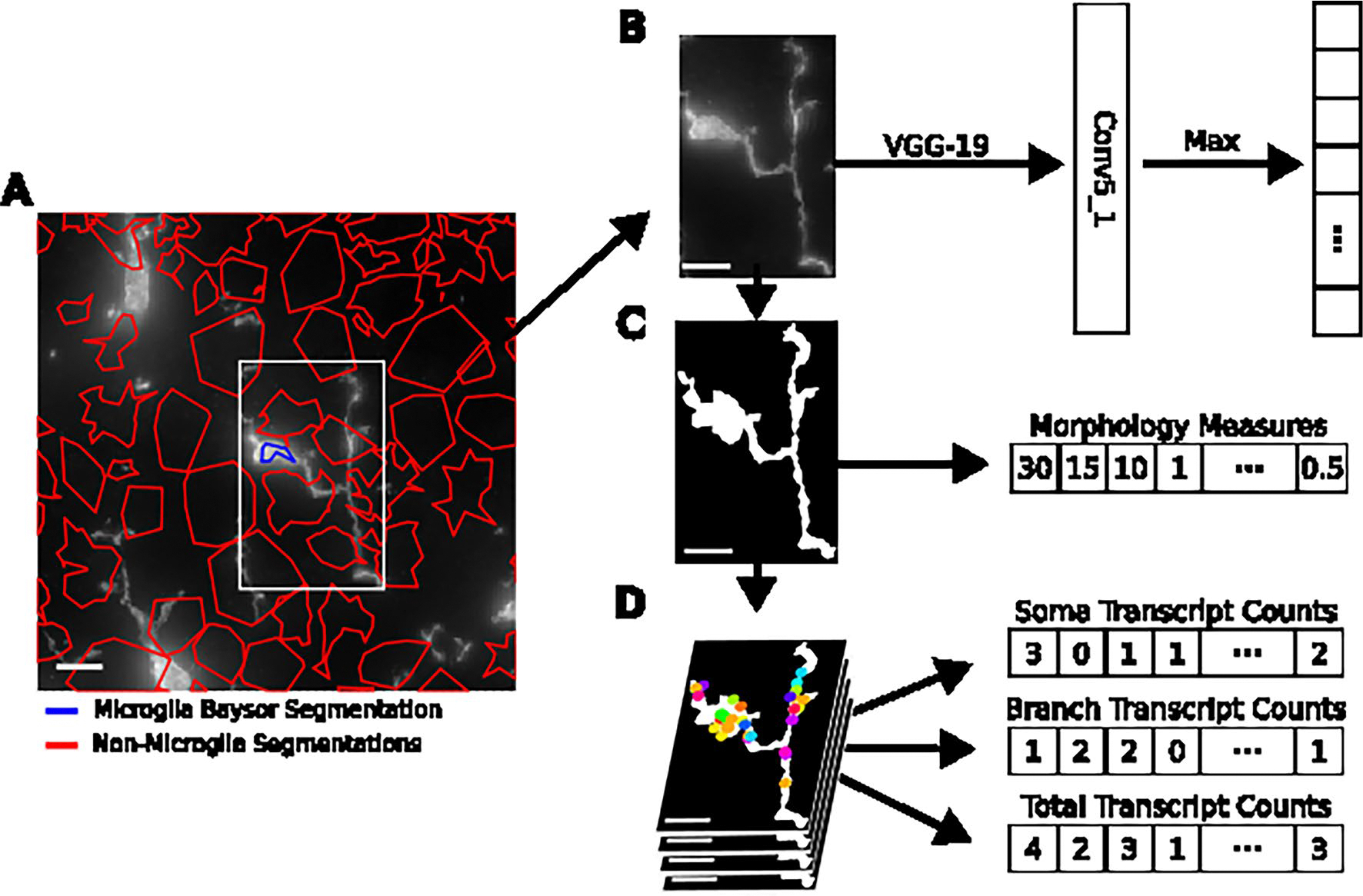
Schematic of Analysis Pipeline. **A**, Matched Baysor cell segmentations with microglia-stained image, colored by selected microglia (blue) and non-selected (red) cells. **B**, Morphological embedding strategy for raw microglial single cell image. **C**, Representative microglial cell segmentation and schematic representation of manual analysis yielding a morphology vector. **D**, Decoded transcripts overlayed onto microglia morphological rendering for multiple z-planes, and schematic representation of three separate counts tables generated for each cell. Scale bars are 10 μm.

**Extended Data Fig. 3 |:**
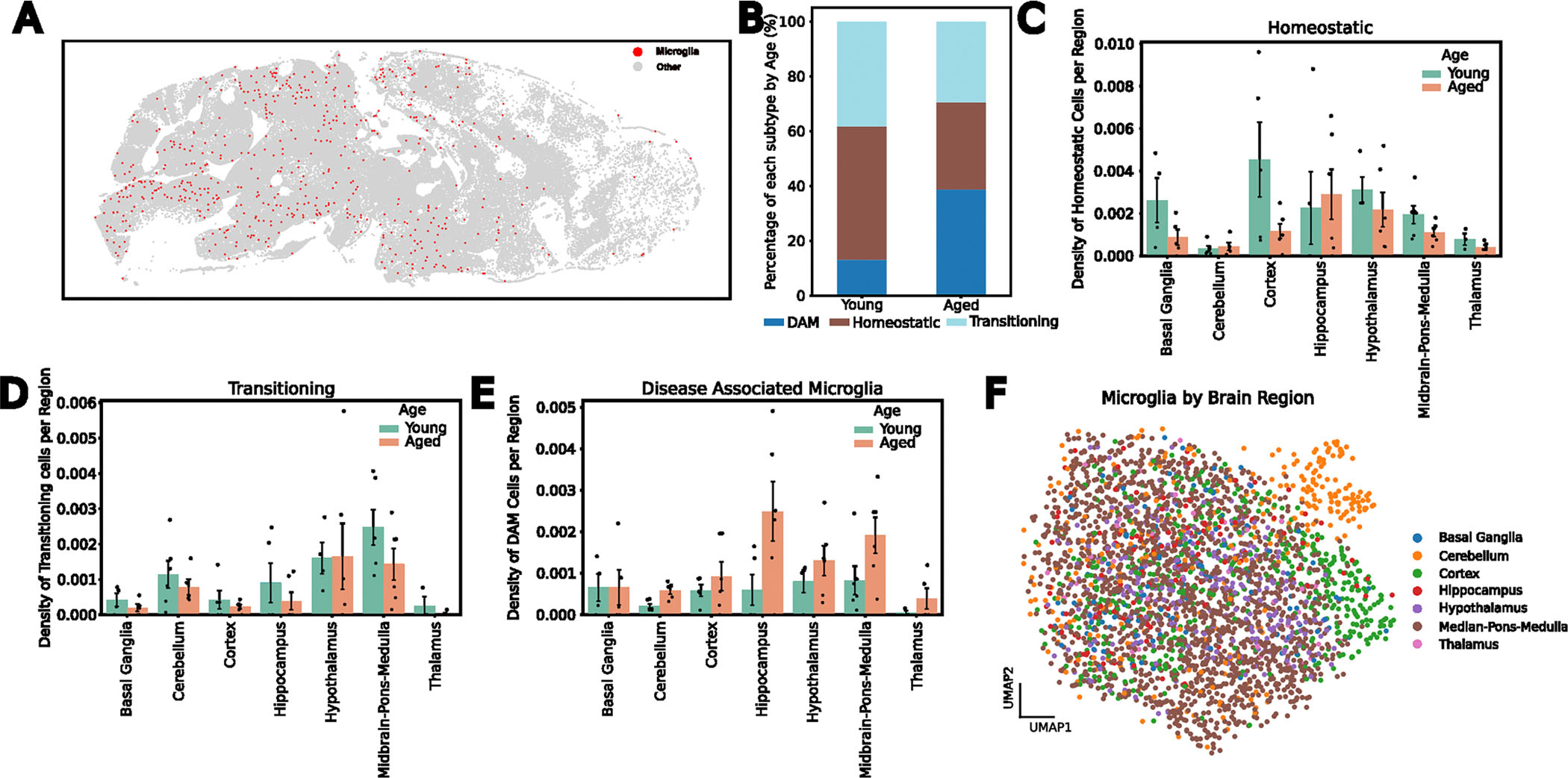
Spatiotemporal Patterning of Transcriptomically Defined Microglia. **A**, Representation of microglia distribution in the sagittal section of a brain. **B**, Frequency of microglial transcriptomic clusters in each age group. **C-E**, Quantification of different microglial transcriptomic clusters distribution across the brain with age, including the Transitioning (**C**), the Homeostatic (**D**), and the DAM-like (**E**) defined microglia. **F**, Transcriptomic UMAP plots of all microglia colored by brain region. All error bars shown are standard error of the mean, and the bar height denotes mean value. The underlying data distributions are shown as bulked density of cells per mouse/brain regions (max points per bar: n = 6).

To characterize microglial regional distribution and transcriptional states with age, we identified and reanalyzed microglia across all 12 brain sections (Methods). After identifying approximately 5,800 transcriptomically defined microglia, we aligned the transcriptomics data to the fluorescent images and used IBA1-based segmentation to refine microglial boundaries (Extended Data Fig. 2a–c). We then recomputed RNA molecule counts within these boundaries to obtain single-cell expression profiles for the microglia (Extended Data Fig. 2d). In total, we identified 3,970 microglial cells (Methods) directly aligned to non-overlapping IBA1 segmentations representing their morphologies. These microglia were predominantly in posterior brain regions, especially the midbrain, pons and medulla (MPM) (Extended Data Fig. 3a). Clustering and two-dimensional uniform manifold approximation and projection (UMAP)^37^ visualization of these aligned microglia revealed three primary transcriptional subtypes (Fig. 1c–e and Extended Data Fig. 3b). The homeostatic cluster, marked by the expression of canonical microglial markers *P2ry12*, *Tmem119* and *Cx3cr1* (ref. 24), was enriched in the cortex (Fig. 1d and Extended Data Fig. 3c). A transitory state of microglia expressed high levels of *Cd83* and *Hif1a*^25^ and was primarily localized to the MPM and the hypothalamus (Fig. 1d and Extended Data Fig. 3d). The third cluster, defined by elevated expression of DAM markers *Itgax*, *Axl* and *Spp1* (ref. 26), was primarily found in the cerebellum, hippocampus and MPM (Fig. 1d and Extended Data Fig. 3e) and was the main subtype increased in aged versus young mice (Extended Data Fig. 3b). We also observed a small region-dependent effect, where microglia in subsets of cortical layers and cerebellum showed slight transcriptomic deviations from other populations (Extended Data Fig. 3f).

**Extended Data Fig. 4 |:**
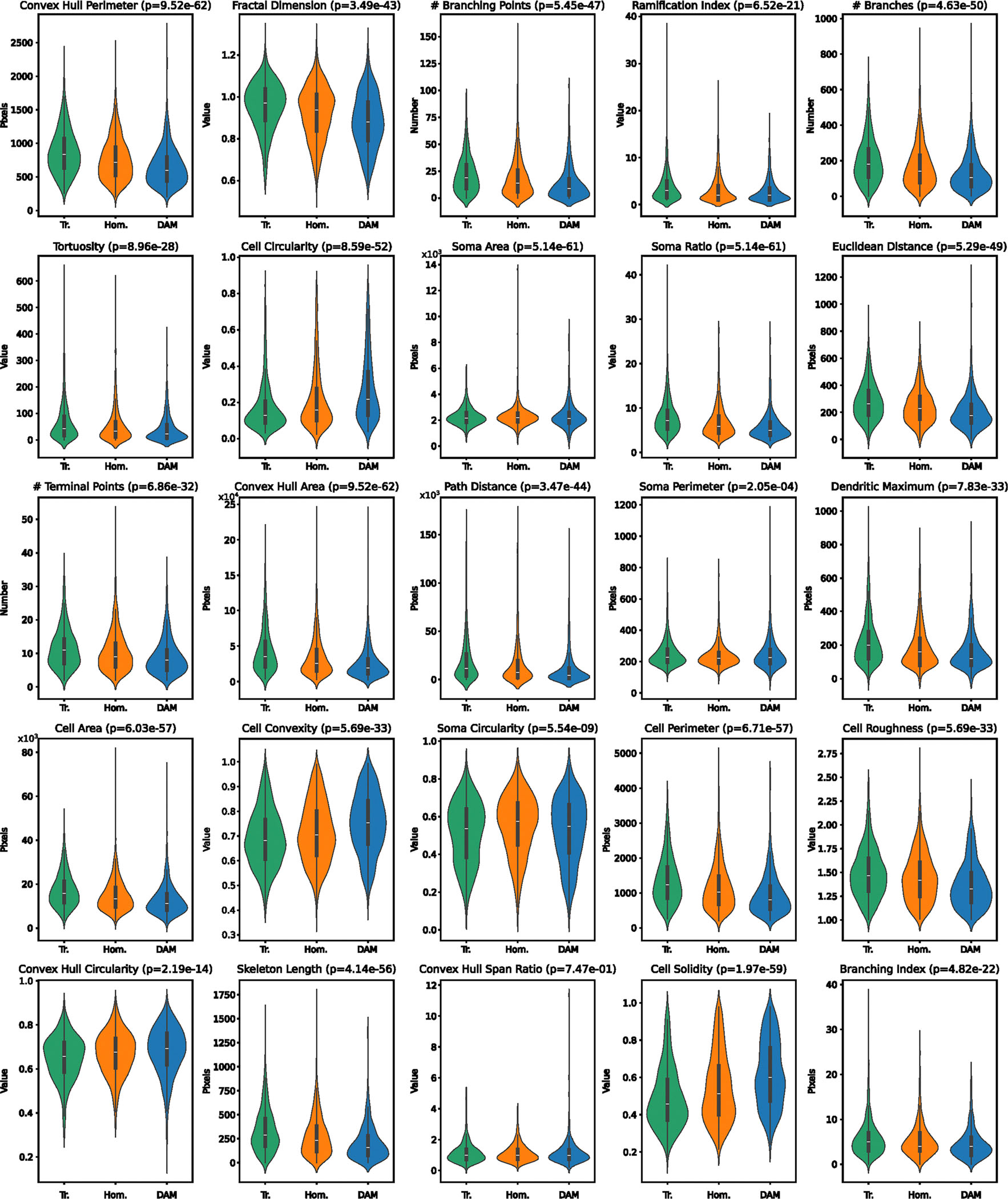
Morphological Characteristics of Transcriptomically Defined Microglia. Selection of quantifications of microglial morphological features, split by transcriptomic cluster. All measurements are either dimensionless, counting the occurrence of a phenomenon, or are pixel-wise quantifications. Samples were compared using an FDR-corrected Kruskal-Wallis test. The corrected p-values are which are displayed as less than 1e^−308^ are reported as such due to numerical precision limits. The boxplot drawn in each violin spans the interquartile range with a central line marking the median. The whiskers extend to the most extreme points within 1.5 times the interquartile range for the upper and lower quartiles. The violin plots were generated from the morphological values of n = 1252 Transitioning microglia, n = 1673 Homeostatic microglia, and n = 1045 DAM-like microglia.

Given that microglial function is typically associated with morphological changes, we next quantified morphological differences across transcriptomic classes. Using the same IBA1 segmentation, we calculated 26 morphological features for all microglia (Extended Data Figs. 2c and 4 and Supplementary Data Table 1). To validate that our thin sections captured intact morphologies, we compared features from whole microglia with those from matched sections of the same cells and observed strong positive correlations (Supplementary Data Fig. 1). Upon confirmation that our morphological measurements reliably reflected the true three-dimensional structure of the cells, we then compared all features between the different transcriptomic classes (Extended Data Fig. 4). As expected, microglia expressing a DAM signature exhibited a more amoeboid morphology, with elevated values of cell solidity (Extended Data Fig. 4) compared to the other two classes on average^38^. Interestingly, both transitioning and homeostatic clusters showed substantial variation in ramification (number of processes), with transitioning cells displaying slightly more branching points. We also observed high morphological variance among DAM-like microglia, with some cells showing ramification similar to homeostatic microglia, contradicting previous assumptions about these states (Extended Data Fig. 4). Together, these findings suggest a disconnect between transcriptomic classification and inferred morphological ramification patterns and point to additional functional heterogeneity within DAM-like microglia based on their morphological properties.

### Microglial functional states defined by transcriptomic states have heterogeneous morphology

**Extended Data Fig. 5 |:**
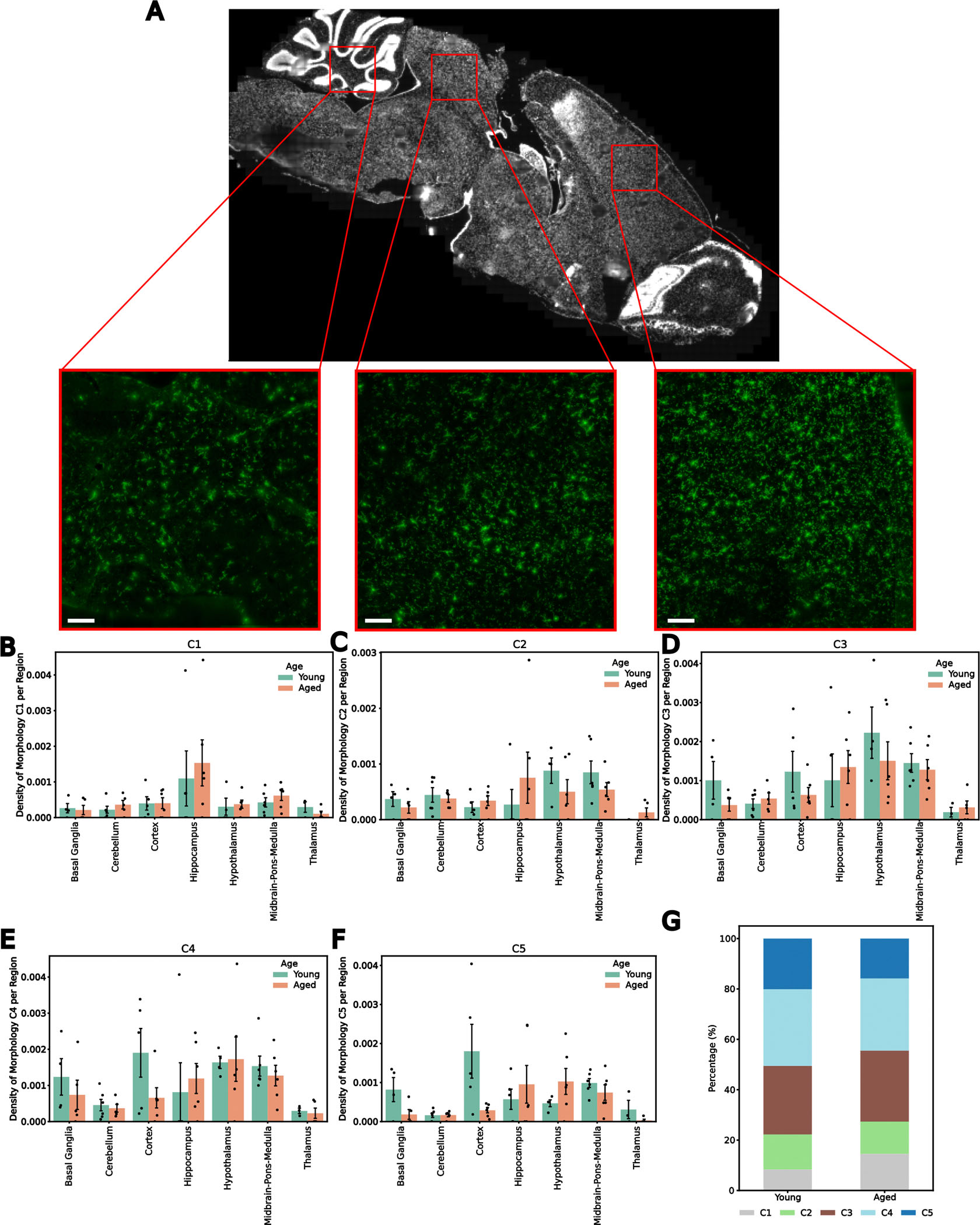
Spatiotemporal Patterning of Morphologically Defined Microglia. **A**, Schematic representation depicting IBA1 stained images as distributed in different regions of the brain. Scale bars are 100 μm. **B-F**, Quantification of different microglial morphological clusters’ distribution across the brain with age, including the amoeboid clusters C1 (**D**) and C2 (**B**) and the ramified clusters C3 (**C**), C4 (**E**), and C5 (**F**). **G**, Frequency of microglial morphological clusters in each age group. The error bars are standard error of the mean, and the bar height denotes mean value. The underlying data distributions are shown as bulked density of cells per mouse/brain regions (max points per bar: n = 6).

**Extended Data Fig. 6 |:**
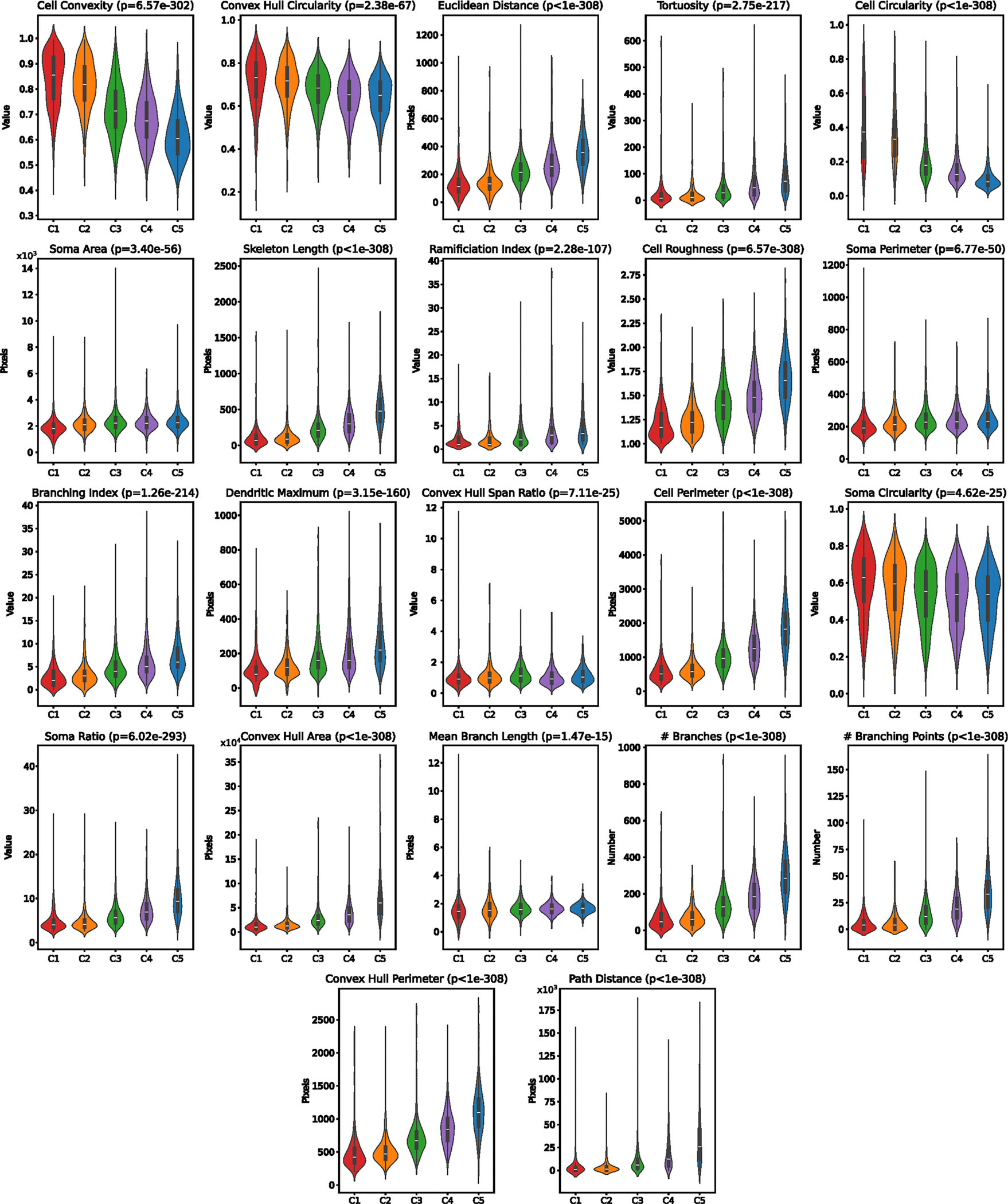
Morphological Features of Morphologically Defined Microglia. Selection of quantifications of microglial morphological features that did not appear in the main text, split by morphological cluster. All measurements are either dimensionless, counting the occurence of a phenomenon, or are pixel-wise quantifications. Samples were compared using an FDR-corrected Kruskal-Wallis test. The corrected p-values are which are displayed as less than 1e^−308^ are reported as such due to numerical precision limits. The boxplot drawn in each violin spans the interquartile range with a central line marking the median. The whiskers extend to the most extreme points within 1.5 times the interquartile range for the upper and lower quartiles. The violin plots for morphological measurements are generated from all 471, 542, 1082, 1157, and 718 cells in clusters C1-C5 respectively.

**Fig. 2 |:**
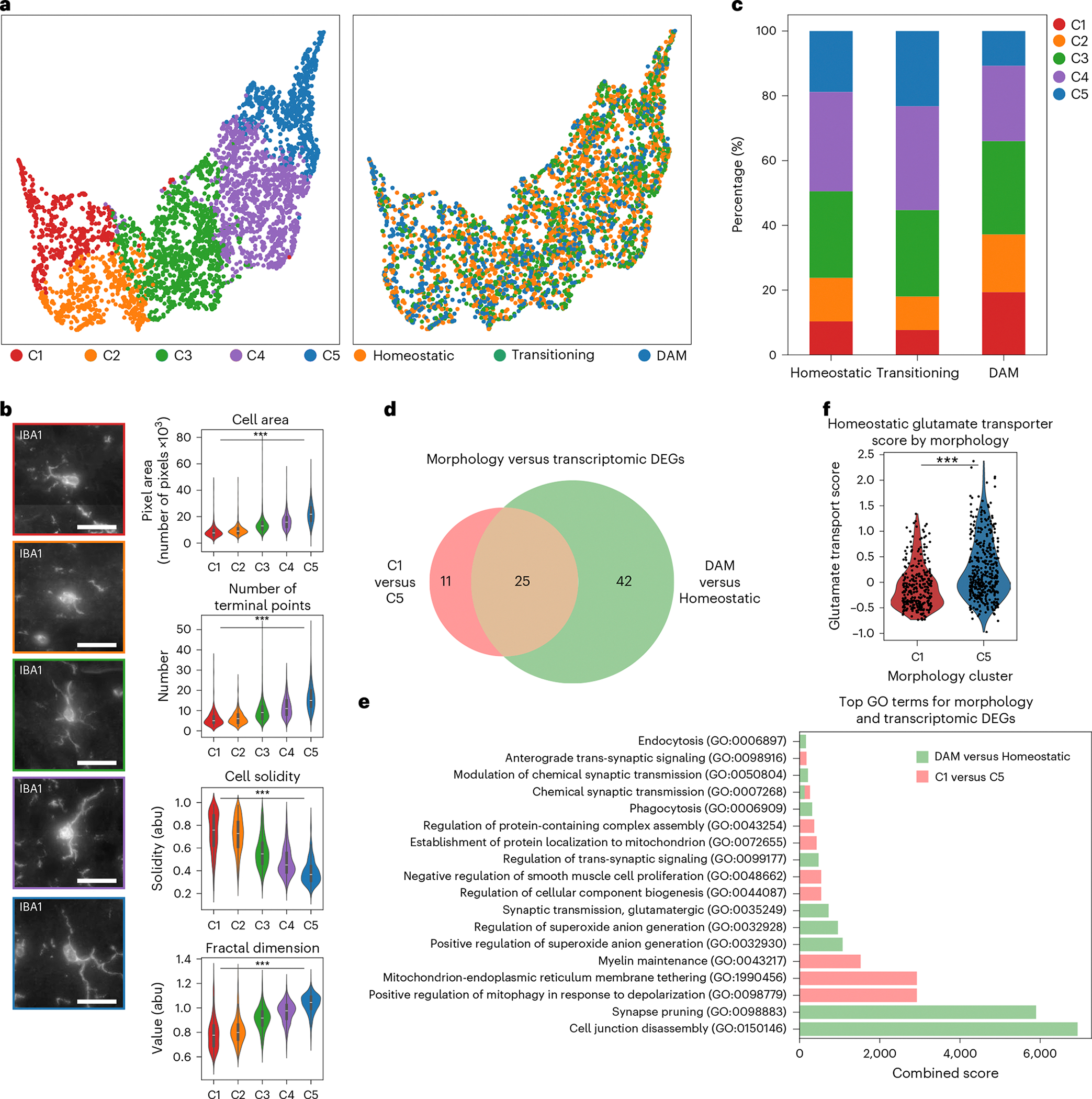
Discrepancies between implied microglia function defined by morphology versus transcriptional state. **a**, Morphological UMAP embeddings of microglia (*n* = 3,970 cells), colored by Leiden clusters (left) and by identified transcriptomic clusters (right). **b**, Representative images of microglia from each of the morphologically defined Leiden clusters (left) and a selection of manually measured morphological features for each Leiden cluster (right). Violin plots for morphological measurements are generated from all 471, 542, 1,082, 1,157 and 718 cells in clusters 1 through 5 (C1–C5), respectively. Samples were compared using an false discovery rate (FDR)-corrected Kruskal–Wallis test. Scale bars, 25 μm. The corrected *P* values for all comparisons shown are less than 1 × 10^−308^ and do not yield an exact *P* value due to numerical precision limits. The box plot drawn in each violin spans the interquartile range with a central line marking the median. The whiskers extend to the most extreme points within 1.5 times the interquartile range for the upper and lower quartiles. **c**, Frequency of morphological Leiden clusters per microglia transcriptomic cluster. **d**, Comparison of DEGs between C1 and C5 and between DAM-like and homeostatic microglia. **e**, Gene Ontology Biological Process terms, sorted by combined score, for each of the comparisons made in **d**. **f**, Comparison of a glutamate transporters scored expression, with a *P* value of 1.53 × 10^−14^ via a Mann–Whitney–Wilcoxon test, between different morphologically classified cells among homeostatic microglia (*n* = 315 C5 microglia; *n* = 173 C1 microglia). ***P < 0.001. GO, Gene Ontology. abu, arbitrary units.

After examining the morphologies associated with different transcriptomic states of microglia, we next asked whether their geometry provided additional insight into functional states. To quantify morphology, we used the aligned IBA1 maximum projection images and Bayesian-defined cell boundary priors to generate single-cell microglial images (Extended Data Fig. 5a). We then used a pretrained neural network to parameterize these images into a high-dimensional morphology embedding that captured multiscale features^39^ (Extended Data Fig. 2b). We then clustered microglia in this space and visualized them using a UMAP embedding (Fig. 2a). Applying *k*-means clustering to the morphology embeddings, we identified five clusters arranged along a continuum from the least to the most ramified morphologies (Fig. 2b). We labeled these C1–C5, with C1 showing the lowest ramification values, such as cell area, number of terminal points and fractal dimension, and C5 showing the highest. Regional distributions recapitulated previous findings where more ramified microglia were enriched in cortex, whereas amoeboid cells were found predominantly in the midbrain and hindbrain^40^ (Extended Data Fig. 5b–f) while increasing with age (Extended Data Fig. 5g)^41^. The morphological classes were characterized by variations in both basic measurements, such as cell area and solidity, as well as ramification metrics, such as fractal dimension and the number of terminal branches^42–45^ (Fig. 2b), confirming that the morphology embedding captured variation in microglial ramification (Extended Data Fig. 6 and Supplementary Data Table 1).

We next compared morphology-derived and transcriptomics-derived clusters by calculating the percentage of each morphology class within each transcriptomic class (Fig. 2a,c). As expected, DAM-like microglia showed reduced ramification, with higher proportions of amoeboid classes C1 and C2 and lower proportions of the ramified classes C4 and C5, whereas C3 was uniformly distributed across all transcriptomic classes. However, we also observed, in contradiction with previous observations, populations of homeostatic microglia with amoeboid morphologies and DAM-like cells with ramified morphologies (Fig. 2c). We validated the presence of these ramified microglia expressing a DAM signature through immunohistochemistry of microglia (Supplementary Data Fig. 2a and Methods), which showed that AXL-positive microglia (microglia expressing a DAM signature) comprised both ramified and amoeboid cells based on the mean morphological values of C4 microglia (Supplementary Data Fig. 2b–d). These ramified DAM-like cells, representing approximately 30% of the transcriptomic class, might have an increased surveillance area compared to their amoeboid counterparts while maintaining their phagocytic and proinflammatory function. Additionally, we see that transitioning microglia displayed the highest proportion of ramified morphologies, suggesting that homeostatic microglial cells display a slight increase followed by a decrease in ramification when undergoing functional responses (Fig. 2c). Thus, a morphological gradient exists across transcriptomic classes, and the pronounced morphological heterogeneity within each class supports the now-accepted concept that function, morphology and transcriptional state are not necessarily connected in the manner once assumed^46^, further calling for the revision of the classic homeostatic–DAM classification framework. The variable proportions of the different morphologies for each transcriptomic class also imply a range of the functional implications that the morphological states can have on the different transcriptomic classes, further skewing previous definitions of microglial cell state.

To further probe relationships between transcriptional state and morphology, we performed differential gene expression analyses between morphology clusters C1 and C5, the extremes of the morphology space, and between homeostatic and DAM-like clusters, the extremes of the microglial reactive states (Fig. 2d). Comparative analysis of the most enriched Gene Ontology Biological Process terms derived from these differentially expressed gene (DEG) sets showed that the transcriptomic changes between homeostatic and DAM-like microglia were associated with Gene Ontology terms such as cell junction disassembly and phagocytosis. By contrast, DEGs between the two extremes of the morphology space were linked to cellular component biogenesis and metabolic processes (Fig. 2d,e), suggesting functional differences between cells of varying geometries that are independent of changes between homeostatic and DAM phenotypes. Together, these findings indicate that reduced ramification does not necessarily correlate with the DAM functional state. Additionally, comparing the morphologically distinct cells within the homeostatic microglia, we observed that genes related to glutamate trafficking and to the anchoring of glutamate receptors, which are associated with ‘activated’ microglia (Methods), are elevated in the ramified microglia as opposed to the amoeboid-shaped cells (Fig. 2f). These results contradict that increased expression of different glutamate trafficking genes results in activated and morphologically less complex cells, as observed previously^47,48^. Collectively, comparing our morphology-based clustering to the transcriptomics-based classification of microglia revealed previously unrecognized heterogeneity, which could not be fully explained through either transcriptomic or morphological analyses alone.

### Gene morphology correlations could contribute to shape heterogeneity

**Fig. 3 |:**
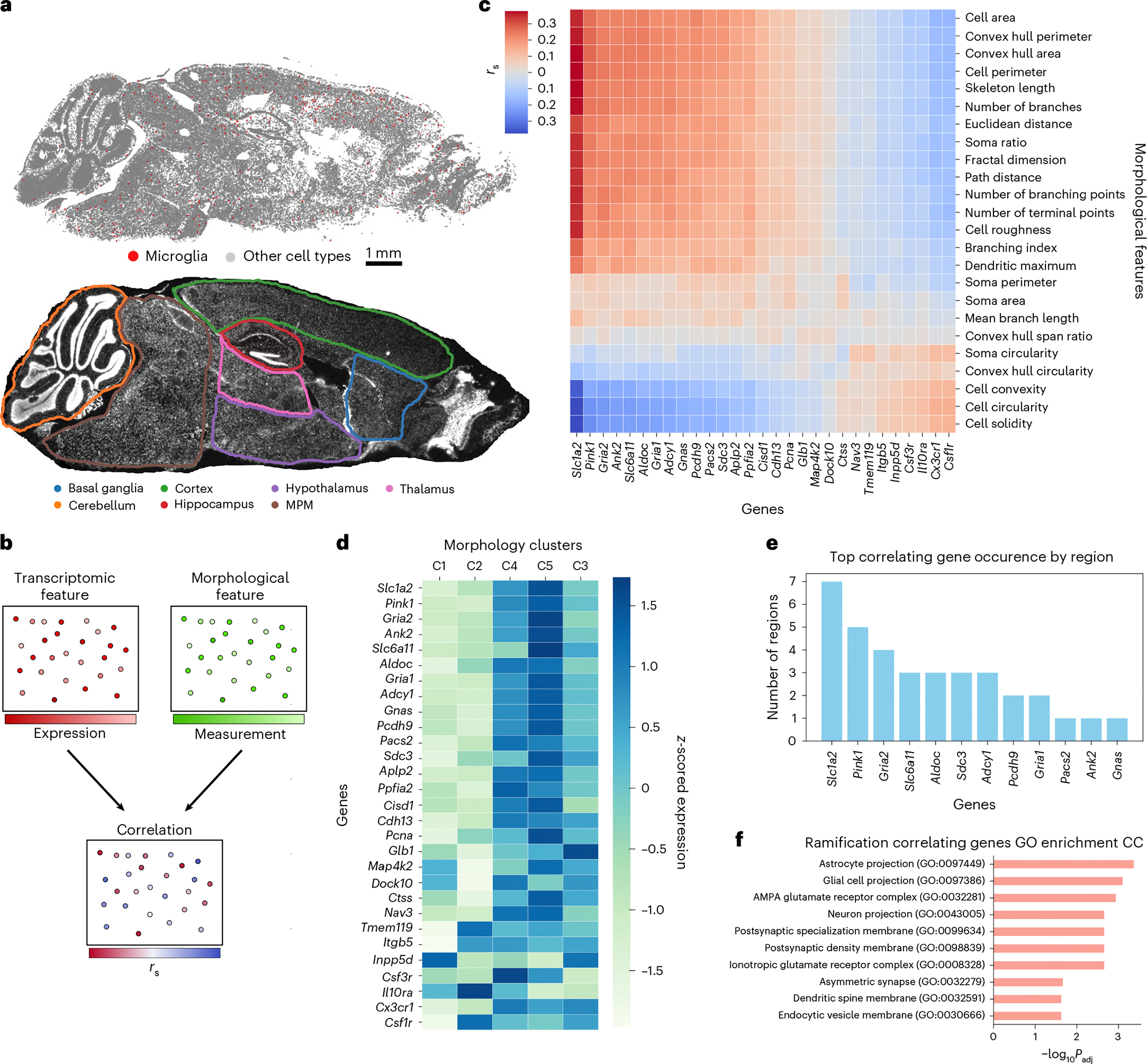
Morphology–transcriptome correlation identifies compartment-enriched genes. **a**, Schematic image of microglia among all other cells in the brain (top) and image of the same brain separated into the brain regions (bottom). **b**, Schematic representation of workflow for establishing correlation results. For every transcriptomic feature, we use a morphological feature on the same microglia to calculate the Spearman correlation (*r*_s_). **c**, Clustered heatmap showing the Spearman correlation between different microglial morphological features and select genes. **d**, Heatmap showing the ability of select genes to be able to cluster the cellsʼ morphological classifications. **e**, Genes among the top correlating genes for morphology characteristics in each region of the brain, sorted by number of regions in which they occur. **f**, Gene Ontology cell compartment terms for the genes that are among the top correlating genes in any segmented region of the brain. CC, Cellular Component; GO, Gene Ontology.

Given the observed disconnect between transcriptomic states and morphology, we next asked how morphological features relate to gene expression. Previous work suggested links between microglial gene expression and ultrastructure^33^. To test for dependencies between cellular morphology and the transcriptome, we performed Spearman correlations between expression of microglial-specific and ubiquitously expressed genes and morphological features using paired measurements across all brain regions (Fig. 3a–c and Methods). This analysis revealed two distinct clusters of genes and features, identifying candidate markers of microglial morphological status. Interestingly, genes such as *Csf1r* and *Cx3cr1*, which are upregulated in homeostatic microglia, are positively correlated with ‘amoeboid’ morphological features. By contrast, genes such as *Slc1a2* and *Gria2* correlated with ramification features. *Slc1a2* was particularly enriched in cells from the most ramified morphological clusters (Supplementary Data Fig. 3a and Methods). To confirm that these ramification-associated genes are expressed in microglia, as seen in single-nucleus sequencing^49^, rather than reflecting astrocyte contamination, we performed single-molecule fluorescence in situ hybridization (smFISH) on brain tissue. *Slc1a2* and *Gria2* puncta were present within IBA1-positive microglia across ages and ramification states (Supplementary Data Fig. 3b,c). To further exclude astrocyte contamination, we performed dual immunostaining with both the microglial marker IBA1 and the astrocyte marker ALDH1L1, supporting the existence of *Slc1a2* puncta, which co-localized with IBA1 and not with ALDH1L1 (Supplementary Data Fig. 4a,b and Methods).

Using transcriptomic features of morphologically distinct microglia, we curated a gene set that captures variance in both morphology and transcriptomic space. We combined the top five genes correlated with each morphological property, yielding 29 morphology-associated genes. Notably, hierarchical clustering of a held-out population of cells using this gene set separated the cells into two distinct groups, containing distinct morphology clusters defined previously (Fig. 3d). Clusters C3–C5 corresponded to genes associated with ramified morphology, whereas clusters C1 and C2 were associated with genes linked to a more amoeboid morphology (Fig. 3d). These results clearly identify a set of genes that can be used to cluster microglial cells with distinct morphological properties.

**Extended Data Fig. 7 |:**
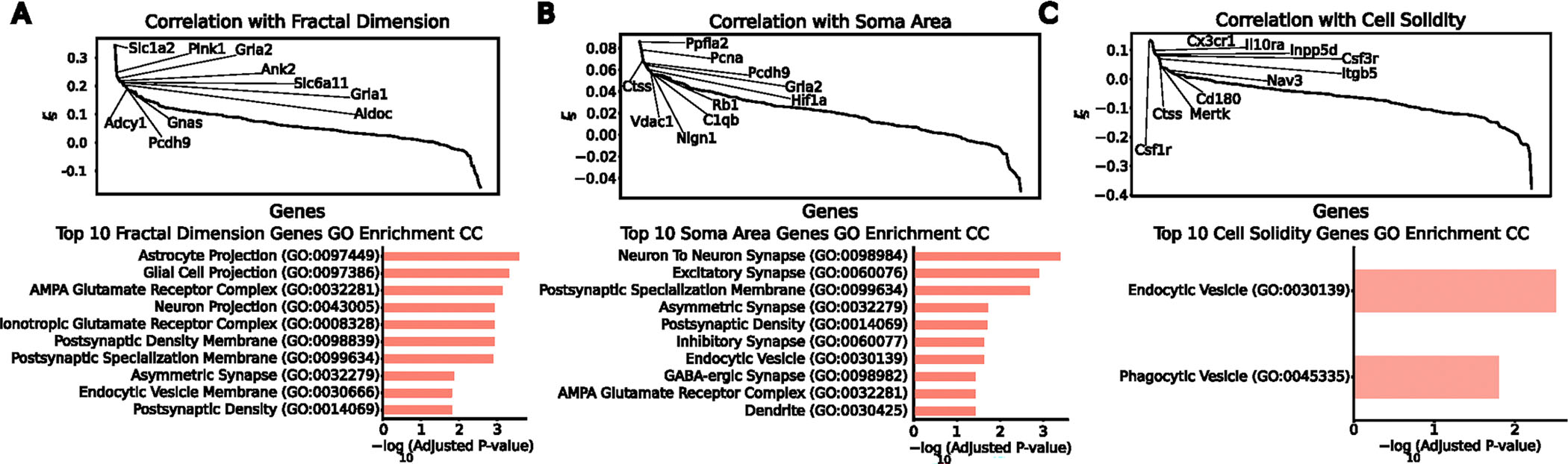
Single Morphological Characteristics Correlate to Compartment Relevant Genes. **A-C**, Rank plots of Spearman correlation between selected morphological features and genes, top 10 genes for each feature are listed (top) and gene ontology cell compartment enrichment for the top 10 correlating genes for each morphological feature. The features selected include the Fractal Dimension (**A**) the Soma Area (**B**) and the Cell Solidity (**C**).

Individual morphological measurements also correlated with genes functionally relevant to the compartments from which those measurements derive (Extended Data Fig. 7a–c). For instance, fractal analysis, which is commonly used to assess microglial complexity^50^, largely relates to the complexity and branching of microglial projections. Genes with the highest correlation to a cell’s fractal dimension were enriched for cell compartment Gene Ontology terms related to morphological projections (Extended Data Fig. 7a). Similarly, genes with the highest correlation to soma area, which is a characteristic of activated microglia that play an active role in inflammation^51^, are enriched for Gene Ontology terms that are consistent with endocytic potential (Extended Data Fig. 7b). Interestingly, cell solidity, a metric canonically associated with amoeboid shape and, therefore, phagocytotic potential^52^, was correlated with genes enriched for both the endocytic and phagocytic vesicle Gene Ontology terms (Extended Data Fig. 7c).

Because the brain exhibits regional heterogeneity in response to inflammatory stimuli^53,54^, we next asked whether regional context modulates the relationship between gene expression and ramification. For each of the seven brain regions in our sagittal MERFISH dataset (Fig. 3a), we calculated Spearman correlations between genes and ramification features (Methods) and compiled the top five genes with the highest average correlation per region (Fig. 3e). Interestingly, *Slc1a2*, which codes for the glutamate transporter 1 (GLT-1) protein, appeared among the top ramification-correlated genes in the largest number of regions. The genes identified in this region-specific analysis were again linked to functional cellular compartments, where those associated with ramification across regions were enriched for Gene Ontology terms related to glial cell projections (Fig. 3f). Collectively, these findings indicate that expression of genes linked to distinct subcellular compartments can effectively stratify microglia by morphology.

### Subcellular enrichment of mRNA varies between genes

**Fig. 4 |:**
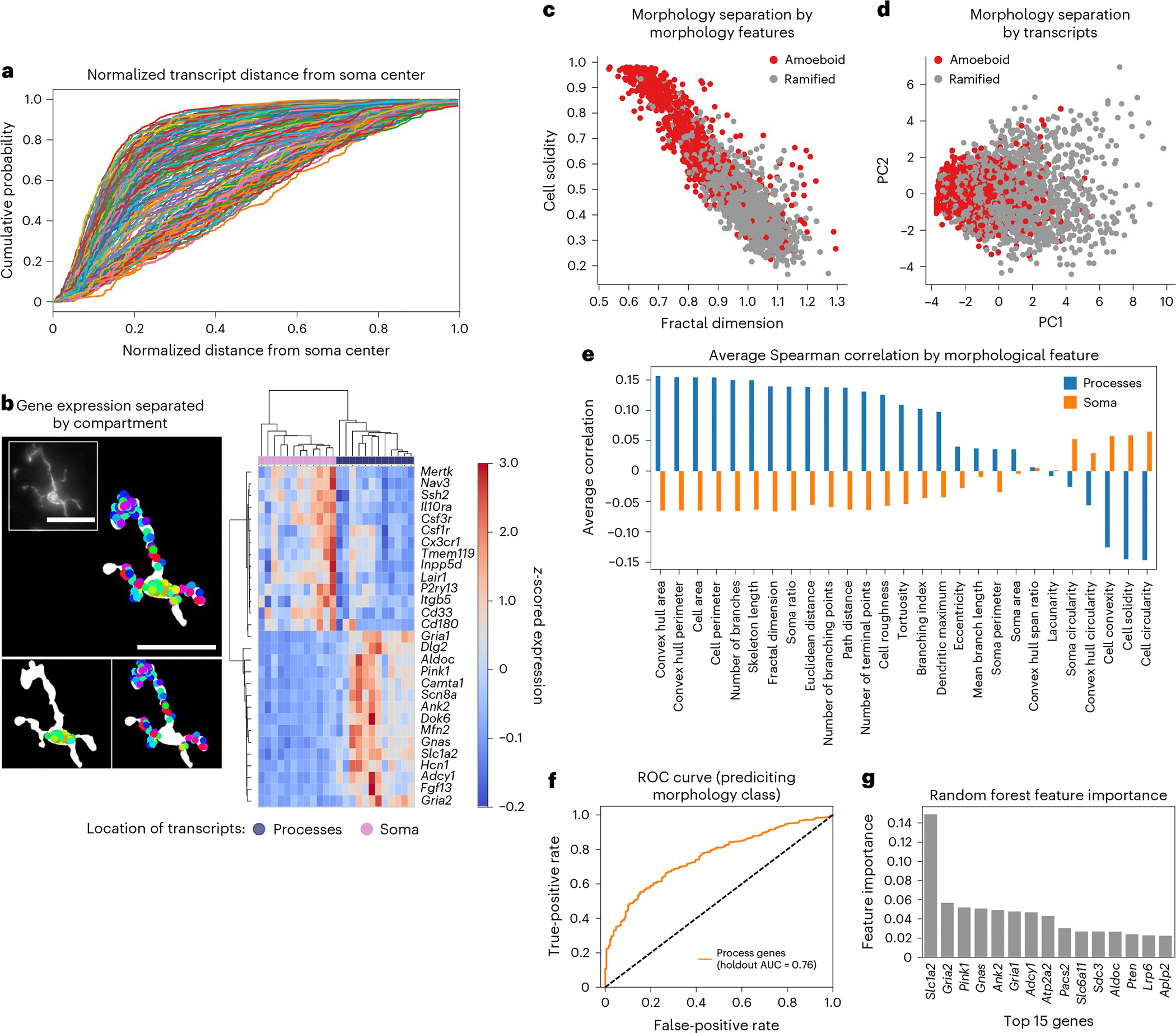
Compartment-enriched genes can predict cell morphology. **a**, The cumulative probability of transcript distance to the soma, normalized for maximum distance within each C4 and C5 cell. Different colored lines represent different genes. **b**, Schematic demonstrating the separation of microglia into soma counts and process counts (left) and clustermap showing that genes cluster by location of transcripts (right). Inset: raw image of the cell. Scale bars, 25 μm. **c**, Amoeboid and ramified cells separate based on morphological features. **d**, Amoeboid and ramified cells separate based on the first and second principal component of compartment-enriched genes. **e**, Average correlation between branch-enriched and soma-enriched genes with morphological characteristics, colored by compartment that genes were enriched in. **f**, Morphological classifier performance as quantified by ROC curve. **g**, Importance of individual genes in classifier as calculated through mean decrease in impurity. PC, principal component; AUC, area under the curve.

The subcellular localization of mRNAs influences functional differences in both neuronal^6^ and glial^55^ cell types. Because genes that correlate strongly with microglial ramification features map to Gene Ontology terms related to glial projections, we asked how transcript distribution across subcellular compartments relates to morphology. We then quantified the normalized distance of each transcript from the soma center in the two most ramified microglial clusters (C4 and C5) (Fig. 4a and Methods). Homeostatic microglial marker genes such as *Cx3cr1*, *Csf1r* and *Tmem119* were enriched closer to the soma, whereas genes such as *Apoe* and *Slc1a2* displayed a more uniform distribution from the nucleus out toward the periphery of the processes, suggesting compartment-enriched mRNA organization in ramified microglia. To more directly examine this, we computationally segmented microglia into soma and process compartments and quantified RNA abundance of microglial-specific and ubiquitously expressed genes in each (Fig. 4b and Extended Data Fig. 2d).

**Extended Data Fig. 8 |:**
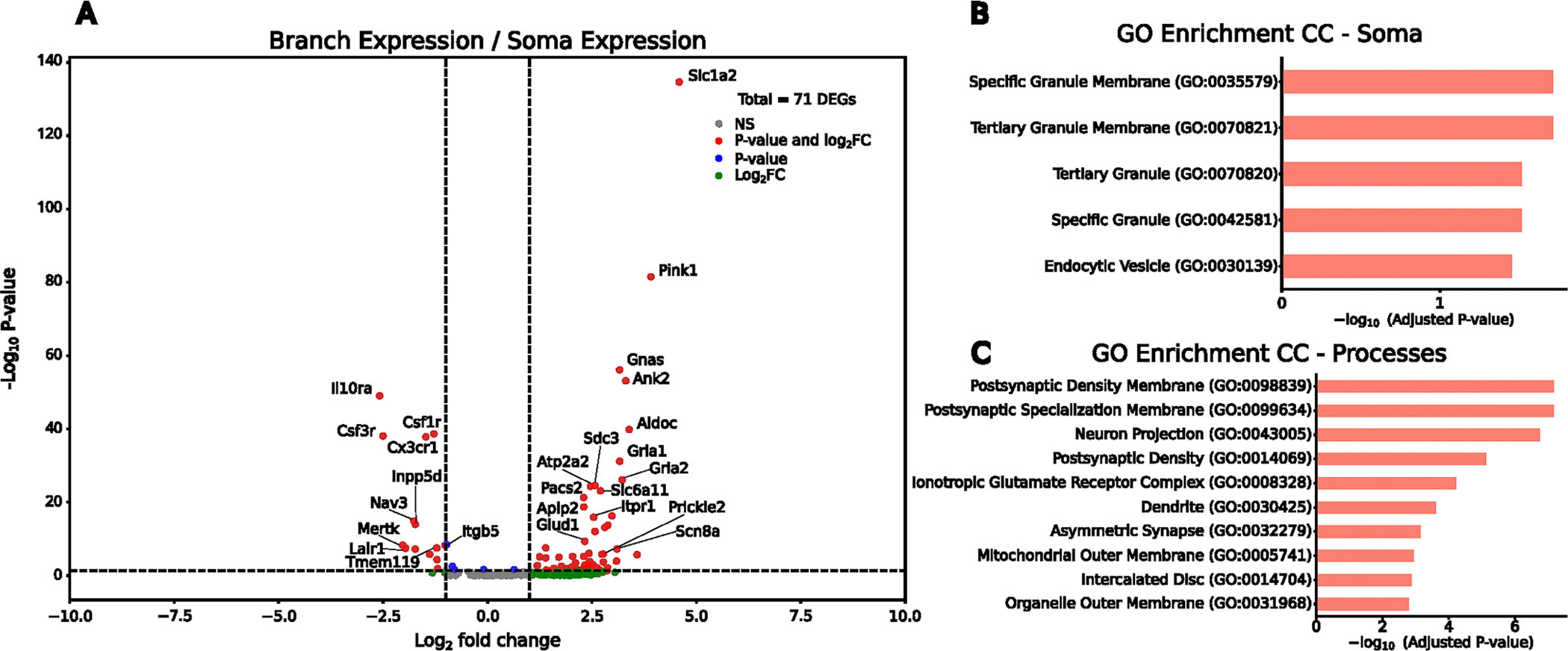
Soma and Branch Specific Transcripts are Enriched for Compartment Relevant Genes. **A**, Quantification of compartment enriched for each mRNA species in the panel. P < 0.05, adjusted P value by Mann–Whitney–Wilcoxon test. FC, fold change; NS, not significant. **B**, Gene ontology terms for cell compartment, calculated from the genes which were enriched in the soma. **C**, The same plot as in **B**, but the gene ontology terms were calculated from the branch enriched genes.

We then compared soma-enriched versus process-enriched transcripts within C4 and C5 to identify genes potentially involved in microglial projections (Methods). From this analysis, we found 14 soma-enriched genes and 57 process-enriched genes (Extended Data Fig. 8a and Supplementary Data Table 2), which clustered by segmented soma or process expression (Fig. 4b). Gene set enrichment showed that soma-enriched genes associated with endocytic vesicles and granule membranes, whereas process-enriched genes were linked to mitochondria and branch-like structures (Extended Data Fig. 8a–c), consistent with compartment-specific functions.

Recognizing that the genes that could be correlated with ramified morphology encode terms reflecting projection cellular compartments, we hypothesized that compartment-enriched gene expression might help to distinguish microglial morphology states. To test this, we grouped microglia into ramified (C4 and C5) and amoeboid (C1 and C2) subpopulations (Fig. 4c) and performed principal component analysis on compartment-enriched genes from the C4 and C5 clusters (Methods). Using whole-cell log-normalized counts from both ramified and amoeboid cells, the first two principal components modestly separated morphological classes (Fig. 4d). To further assess classification power, we computed the average Spearman correlation between each compartment’s enriched gene set and morphological features. Process-enriched genes showed higher average correlations than soma-enriched genes (Fig. 4e), indicating that process-localized transcripts better capture morphological phenotype.

To evaluate the predictive power of process-enriched genes, we trained a random forest classifier using whole-cell expression of process-enriched genes to distinguish ramified from amoeboid microglia. Training on 80% of the microglial cells and evaluating on a held-out set, the classifier achieved a reasonable accuracy, with an area under the receiver operating characteristic (AUROC) value of 0.76 (Fig. 4f). Among the most informative genes were *Slc1a2* and *Pink1* (Fig. 4g), consistent with previous findings that both genes are related to ‘activated’ microglial states and function^47,56,57^. The strong contribution of these process-enriched genes, together with increased *Slc1a2* puncta in more ramified cells (Supplementary Data Fig. 3b,c), indicates that the transcriptional profile of a microglial cell is closely tied to the ramification of its processes. These results support the notion that subcellular transcript distribution plays a key role in shaping microglial morphology and differentially influences microglial functions depending on where transcripts reside.

### Compartmentalization of transcripts may be altered upon aging

The compartmentalization of cellular functions within the brain is thought to be influenced by aging^58^. Because process-localized transcripts effectively predict microglial morphology, we next examined how age affects transcript localization and the ability to infer morphology from compartmentalized gene sets.

**Extended Data Fig. 9 |:**
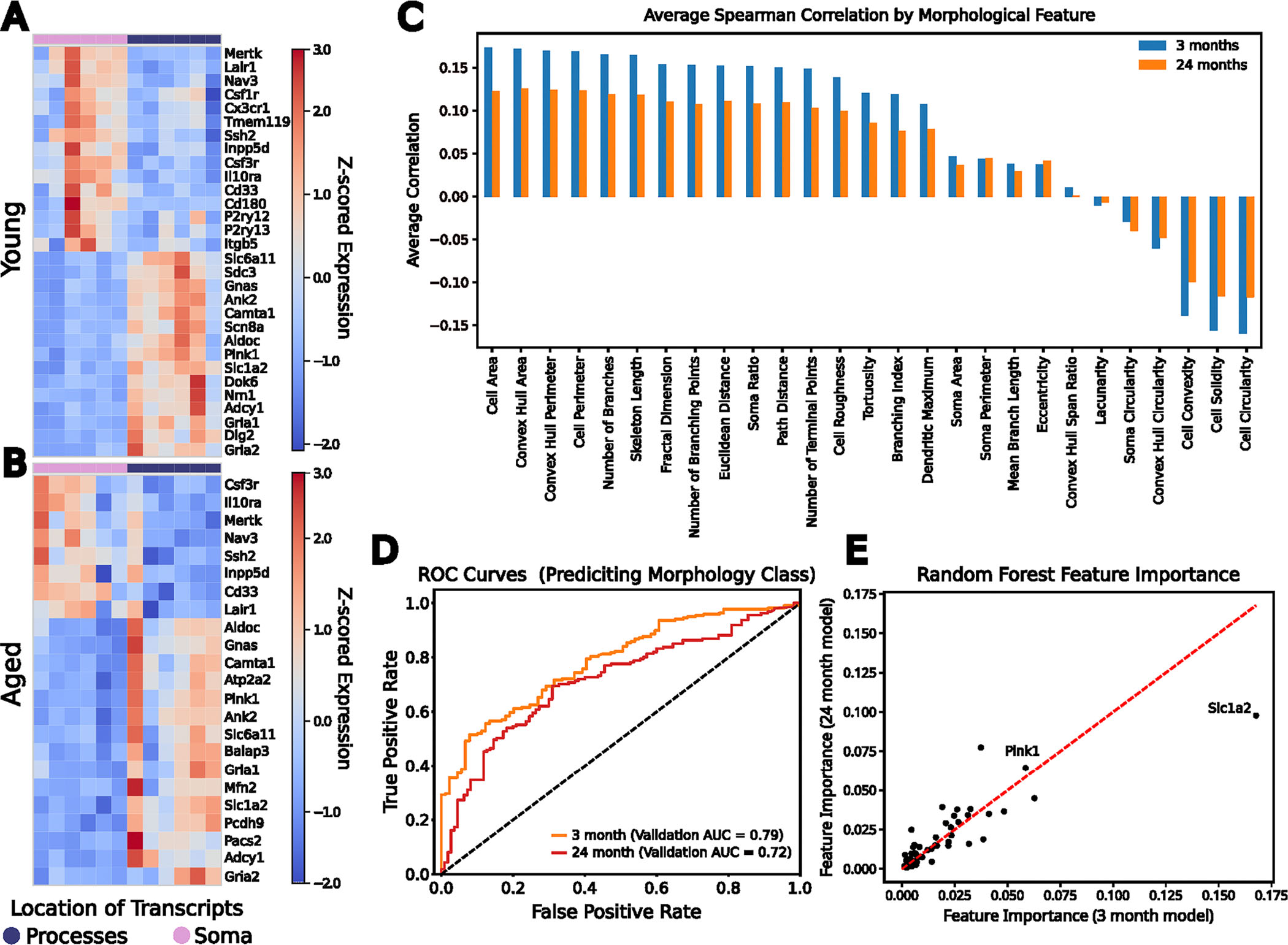
Compartment Enriched Genes Are Affected by Age. **A**, Heatmap showing genes cluster by location of transcripts in young mice. **B**, Heatmap showing genes cluster by location of transcripts in aged mice. **C**, Average correlation between process enriched genes with different morphological characteristics at each age, colored by age of mouse. **D**, Classifier performance as quantified by receiver operator characteristic curve (ROC) for samples from young or old mice. **E**, Importance of individual genes in each age stratified classifier as calculated through mean decrease in impurity.

We compared soma-enriched and process-enriched transcripts in the two most ramified microglial clusters at young and aged timepoints (Methods). In young microglia, we identified 16 soma-enriched genes and 50 process-enriched genes, whereas aged microglia showed only 8 soma-enriched genes and 23 process-enriched genes, and both sets effectively clustered according to compartment-derived gene expression (Extended Data Fig. 9a,b and Supplementary Data Table 2). This reduction in enriched genes in aged cells suggests diminished mRNA compartmentalization.

We then asked whether process-enriched genes retained morphology-predictive power across age. Using the previously defined process-localized gene set, we calculated average Spearman correlations between gene expression and morphological features in young and aged microglia and found stronger correlations in young cells (Extended Data Fig. 9c). To further test the predictive capacity of process-enriched genes across age, we trained random forest classifiers using the process-enriched gene expression profiles in young and aged microglia, each model trained on an 80% random sample of that age group. The resulting models achieved AUROC values of 0.79 for young microglia and 0.72 for aged microglia, reflecting stability in predictive power with age (Extended Data Fig. 9d).

Finally, we examined the key drivers of classification across ages by calculating the relative importance of each gene in the classifiers. This analysis again highlighted *Slc1a2* and *Pink1* as principal factors distinguishing morphological states in both age groups (Extended Data Fig. 9e). Together, these findings suggest that aging reduces the extent of RNA compartmentalization, yet a core set of functional process-enriched genes, including *Slc1a2* and *Pink1*, remains central for defining microglial morphological states.

### Subcellular transcript co-localization changes with age

**Fig. 5 |:**
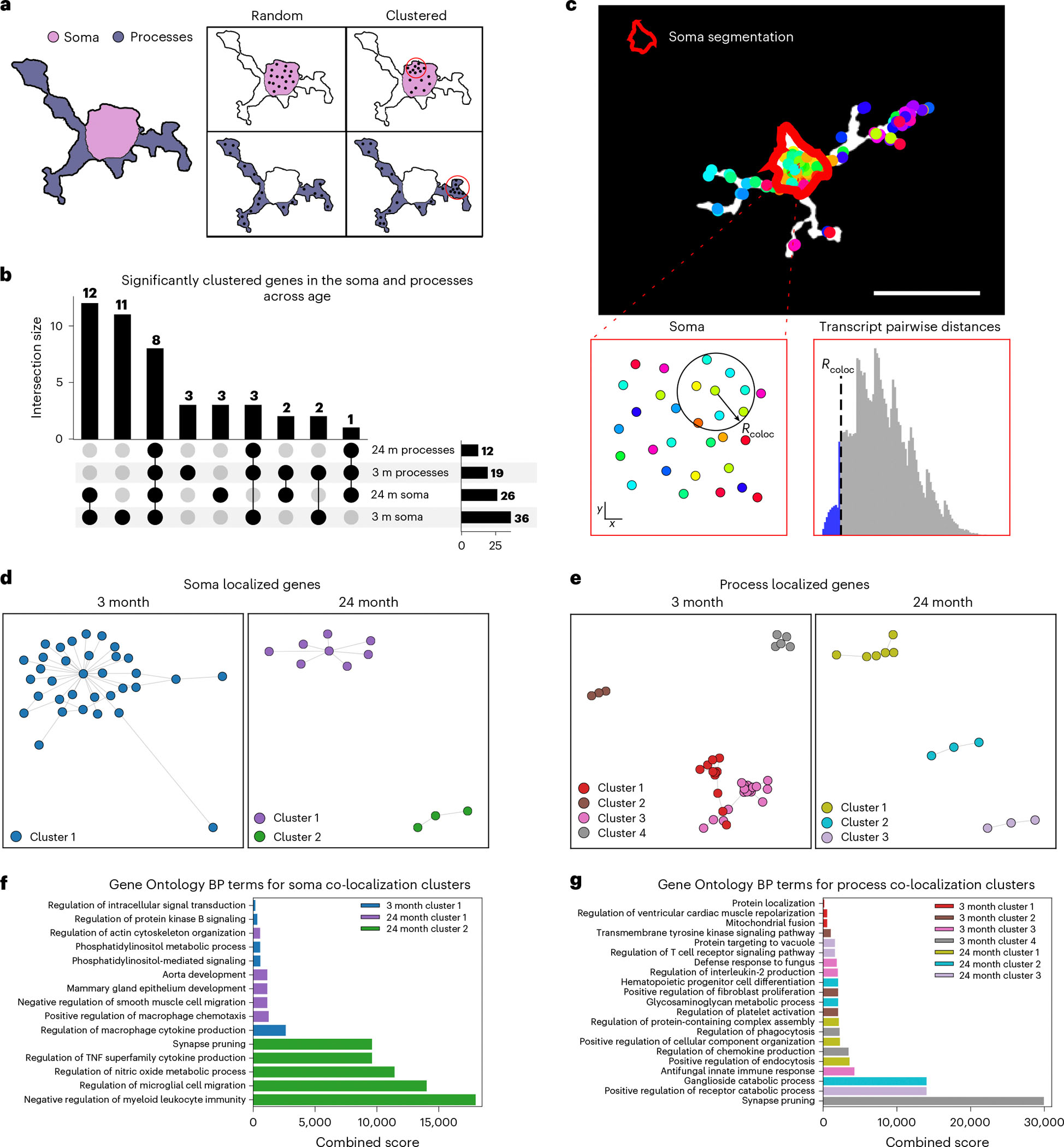
Gene co-localization within microglial soma and processes changes with age. **a**, Schematic comparison between completely random spatial null distribution and spatially clustered sample for both the processes and the soma. **b**, UpSet plot displaying differences in genes that display significant clustering between compartments and ages. **c**, Schematic demonstrating identification of gene–gene pairs with a co-localization threshold *R*_coloc_ (left) and schematic for how the *R*_coloc_ was set (right). Scale bar, 25 μm. **d**, Statistically significant soma-specific gene–gene localization networks for both young (left) and aged (right) microglia. The color in both graphs reflects Gaussian mixture model clustering of the individual networks. **e**, Statistically significant process-specific gene–gene localization networks for both young (left) and aged (right) microglia. The networks are colored by Gaussian mixture model clustering. **f**, Significant Gene Ontology Biological Processes across both young and old soma-specific gene localization networks. The colors reflect the spectral clustering colors defined in **d**. **g**, Significant Gene Ontology Biological Processes across both young and old process-specific gene localization networks. The colors reflect the spectral clustering colors defined in **e**. BP, Biological Process; GO, Gene Ontology; m, months.

The compartmentalization of mRNA, and its resulting functional impact, appears to shift with age. Previous work showed that functional differences arising from mRNA compartmentalization are influenced not only by the localization of transcripts but also by their spatial proximity to one another^59–61^. Given that aging has been shown to affect mRNA compartmentalization within the brain^62–64^, we next examined how it influences the spatial clustering of mRNAs within specific subcellular compartments. To quantify clustering, we applied a generalized Ripleyʼs *K* analysis, a method designed to assess transcript clustering against a spatially randomized distribution^65–67^ (Methods). Because we had observed that expression patterns vary between soma and processes with age, we conducted separate clustering analyses for each compartment in the C4 and C5 cells (Fig. 5a). Our results showed that the age-related changes in mRNA clustering differed by compartment (Fig. 5b), implying that aging may drive different functional modifications for each compartment.

We hypothesized that such age-dependent shifts in mRNA clustering could affect function by altering networks of co-localized transcripts^68,69^. To test this, we first calculated pairwise distances between all transcripts localized to the soma and used the bottom fifth percentile of these distances as our co-localization threshold (Fig. 5c). For each pair of genes, we quantified the number of corresponding transcripts that fell within this threshold. To evaluate whether the observed co-localization exceeded random expectations, we generated null distributions by randomizing RNA positions within the segmented soma and process compartments and recalculating co-localization across 1,000 randomized cells (Methods).

This approach revealed significant age-related reductions in mRNA co-localization. In the soma, 37 gene pairs showed significant co-localization in young microglia versus only nine in aged microglia. Similarly, in the processes, 35 gene pairs were co-localized in young cells compared to just nine in aged cells (Fig. 5d,e and Supplementary Data Table 3). Clustering these gene networks (Fig. 5d,e and Methods) identified functional gene sets that differed by both compartment and age (Fig. 5f,g).

In the soma, younger mice (3 months old) displayed differences in the functional gene clusters compared to the aged mice (24 months old). These younger clusters were enriched for Gene Ontology terms related to cytokine production and metabolic processes. By contrast, the aged soma network was associated with Gene Ontology terms related to chemotaxis and synapse pruning (Fig. 5f). Turning to the processes, we found that aging also reshaped the functional composition of co-localized gene networks. In young mice, process-enriched networks were associated with protein localization and synaptic pruning, whereas, in aged mice, these networks were linked to endocytosis and regulation of catabolic processes (Fig. 5g). These findings align with previous studies demonstrating that age-related changes in mRNA co-localization can influence downstream brain functions^70^. Taken together, our results underscore how mRNA compartmentalization underlies age-associated shifts in microglial function, with distinct compartment-enriched transcript interactions driving differential biological outcomes.

## Discussion

The spatial organization of transcripts within cells provides crucial insights into their functional states^71^, yet this relationship has remained largely unexplored in microglia. Morphological classification by fluorescence imaging has long been the standard for identifying ‘activated’ cells in subclinical and clinical models^72,73^, but how transcript localization relates to morphology has been unclear. The paradigm that morphology is intertwined with biological function underlies frameworks that infer the transcriptomic landscape of large tissue regions from histological images^74^. Despite this, these inferences had not been investigated at the level of the individual cell or its subcellular compartments. In the present study, we used MERFISH paired with fluorescence immunohistochemistry to simultaneously analyze the morphological and transcriptomic landscapes of individual microglia, including the spatial distribution of transcripts within cellular compartments. This approach revealed heterogeneity in the morphologies of different transcriptomically defined microglia and links these changes to subcellular localization of specific gene sets. We also uncovered discrepancies between microglial morphological and transcriptional states and age-related changes in mRNA compartmentalization, allowing us to identify process-enriched gene sets that classify the ramification status of different cells.

The longstanding doctrine in microglial biology is that ‘activation’ is accompanied by branch retraction, although a recent consensus report has begun to challenge this view^46^. Using high-resolution imaging and transcriptomic profiling of individual microglia, we instead found substantial morphological heterogeneity within transcriptomic subtypes, contradicting this model. Notably, the glutamate transporter gene *Slc1a2*, whose transcripts predominantly localize to microglial processes, is upregulated in more ramified microglia compared to amoeboid cells. Increased expression of GLT-1, the protein encoded by *Slc1a2*, has been linked to ‘activated’ microglia^47^, directly opposing the presumed association between ‘activation’ and amoeboid morphology. Non-functional variants of GLT-1 have been associated with the loss of microglial branching^75^, further supporting our findings. Moreover, amoeboid microglia do not show differential regulation of phagocytosis or synaptic clearance-related genes compared to ramified cells within the same transcriptomic class. Together, these results indicate that transcript localization is a critical determinant of microglial function, partially uncoupled from morphology, and suggest that spatial transcriptome regulation^76^ could be leveraged to correct age-related compartmentalization defects and modulate cellular motility and phagocytic capacity.

Disruption of microglial morphology has been viewed both as an ‘activation’ marker and a consequence of aging^27^. Aging both induces structural changes in the CNS and disrupts functional compartmentalization of RNA^5^. We observed that young microglia have more process-localized genes and distinct gene regulatory networks than aged microglia. These shifts in RNA compartmentalization may arise from altered RNA trafficking^77^ and could represent a protective strategy against age-related degradation of mRNA in the cell body^1,78^. However, process-localized genes remained predictive of morphology across ages, suggesting that aging narrows the breadth of transcript localization but preserves core pathways governing cellular architecture. Defining these conserved mechanisms may reveal strategies to sustain cellular function despite age-related decline in RNA trafficking systems.

Although our approach advances identification of compartment-enriched gene expression, potential technical limitations warrant consideration. Imaging-based spatial transcriptomics provides high-confidence mRNA quantification^79^ but, like other spatial technologies, is constrained by accurate cell segmentation. Despite strict segmentation protocols (Methods), the complex morphology of brain cell types presents inherent challenges. Our GFAP-based subtraction strategy cannot fully eliminate astrocyte contamination, as GFAP immunolabeling does not capture the full territorial extent of astrocytes^80^. Consequently, some genes identified as astrocyte-enriched may reflect residual astrocytic signal, potentially leading to alternative interpretations of compartment-specific expression patterns. We mitigated this by filtering genes specifically expressed in other cell types and validating microglial identity through functional gene signatures, but future work would benefit from segmentation-free strategies, such as proximity labeling^81^, for identifying compartment-enriched gene expression.

As these challenges are addressed, spatial transcriptomic technologies are pushing resolution below the single-cell level^82,83^, enabling detailed views of subcellular mRNA organization across tissues^84–86^. These advances will clarify how transcript positioning within compartments underlies morphological complexity and functional specialization. Pairing such technologies with advanced imaging and full-transcriptome measurements will be essential for understanding how subcellular transcript localization shapes cellular heterogeneity and drives functional complexity. Correlational maps like those generated here can help reveal how disrupted transcript localization contributes to structurally manifested disease^87,88^. Our analysis framework extends to other cell type categories, allowing genetic associations with any morphological or subcellular feature identifiable by fluorescence microscopy. Mapping transcript distributions while simultaneously capturing cellular architecture brings us closer to understanding how spatial RNA organization orchestrates cellular function. Applying this methodology across diverse cell types, with expanded gene panels and improved segmentation, will be key to defining the relationships among spatial RNA organization, cellular morphology and their links to function and interaction. Ultimately, integrating subcellular transcript mapping with morphological analysis may reveal therapeutic targets grounded in both cellular structure and the spatial organization of gene expression.

## Methods

### Mice

C57BL/6J male and female mice (stock no. 000664) were purchased from The Jackson Laboratory. Up to five mice were housed per cage under specific pathogen-free conditions in the Wu-Tsai Neurosciences Research Institute Veterinary Service Center at Stanford University School of Medicine. The colony room was maintained on a 12-hour light/dark cycle with food and water provided ad libitum. All animal care and experimental procedures complied with the Guide for the Care and Use of Laboratory Animals of the National Institutes of Health (NIH) and were approved by Stanford University’s Administrative Panel on Laboratory Animal Care.

### MERFISH analysis

#### MERFISH panel selection.

Using a combination of single-nucleus RNA sequencing data with aging and neuroscience literature, we were able to select genes for MERFISH. Our selection criteria for the single-nucleus RNA sequencing data involved identifying cell type marker genes for each cell population using a one-versus-all approach. To do this, we used an internal dataset with single-nucleus sequencing of the cerebellum, and we used a previously published single-nucleus dataset of the mouse hippocampus^53^. We then performed a Mann–Whitney–Wilcoxon test for each gene between the cells within each cell population and all other cells not in that population and corrected the resulting *P* values for multiple hypothesis testing using the Benjamini–Hochberg correction to obtain false discovery rate (FDR)-adjusted *P* values. As previously described^89^, a gene was considered a cell type marker for a specific cell population if (1) it was expressed in at least 30% of cells within a specific population; (2) the FDR-adjusted *P* value was less than 0.001; (3) gene expression in the specified population was at least four-fold higher than the average expression in all cells not in that population; and (4) it was expressed in a fraction of cells within the specified population that was at least two times higher than any other population of cells. We then saved the top five marker genes for each cell type based on the effect size of the log fold change. In addition to these markers, known genes related to age^53^, microglia, astrocytes, oligodendrocytes and neuronal markers^89^ and genes related to subcellular structure from the literature were included, which brought the panel to a total of 500 genes.

#### Tissue processing for MERFISH.

Brain samples for all mice were processed using a fixed-frozen protocol. After anesthetization with 2.5% v/v avertin, transcardial perfusion with 20 ml of cold (4 °C) PBS was followed by perfusion with 30 ml of 4% paraformaldehyde, also maintained at 4 °C. The brains were immediately removed and submerged in 4% paraformaldehyde for an overnight incubation at 4 °C. After incubation, the brains were then placed in 30% sucrose until sinking. Samples were then hemisected and frozen in Optimal Cutting Temperature (OCT) compound using dry ice, after which they were stored at −80 °C until sectioning. All samples possessed a DV200 value of greater than 60% per manufacturer recommendations. Sectioning of these samples was performed along the sagittal plane on a cryostat at −20 °C. Slices between 10 μm and 20 μm in thickness were captured onto Vizgen slides (Vizgen, 10500001) for MERFISH.

#### Sample preparation for MERFISH imaging.

Vizgen slides with single mouse brain sagittal sections were processed according to a fixed-frozen tissue sample preparation MERSCOPE protocol (Vizgen). The slides, upon removal from the cryostat, were washed three times with 1× PBS and placed in 70% ethanol, at which point they were placed in a photobleacher (Vizgen) for 3 hours; after photobleaching, the samples were stored at 4 °C overnight. After incubation overnight, the samples were washed with 1× PBS and covered with Blocking Buffer C Premix (Vizgen, 20300100) and RNase inhibitor (New England Biolabs (NEB), M0314L) for 1 hour. The samples were then placed in a primary antibody staining solution consisting of Blocking Buffer C Premix, 10% RNase inhibitor, IBA1 antibody (Abcam, ab178846) and GFAP antibody (Abcam, ab4674) at a volume dilution of 1:1,000 for 90 minutes. Primary antibody incubation was then followed by three washes with 1× PBS. After washing, the samples were then placed in a secondary antibody staining solution consisting of Blocker Buffer C Premix, 10% RNase inhibitor, anti-rabbit Aux 5 (Vizgen, 20300102) and anti-chicken Aux 9 (Vizgen, 20300106) at a 1:100 dilution for 1 hour. The samples were then washed three times in 1× PBS followed by a 15-minute incubation with 4% paraformaldehyde and an additional two washes in 1× PBS. Following the staining protocol, the samples were then washed one time with Sample Prep Wash Buffer (Vizgen, 20300001) and then washed with Formamide Wash Buffer (Vizgen, 20300002) for 30 minutes at 37 °C. A 500-gene panel mix (Vizgen, 10400003) was then incubated on the tissue samples for anywhere between 36 hours and 48 hours at 37 °C. After hybridization, the samples were washed with Formamide Wash Buffer two times at 47 °C for 30 minutes each. The tissue samples were then embedded in a 4% polyacrylamide gel and were treated using Clearing Premix (Vizgen, 20300003) and Proteinase K (NEB, NC0547027) overnight at 37 °C to digest proteins and lipids in the samples. After the digestion, the coverslips were washed two times with Sample Prep Wash Buffer, stained with a DAPI/PolyT mix for 15 minutes and washed with Formamide Wash Buffer followed by Sample Prep Wash Buffer one final time each before imaging. Finally, the slides were loaded into the MERSCOPE Flow Chamber and imaged at ×20 magnification to identify regions of interest and ×63 magnification to image the probes.

#### MERFISH data processing and quality control.

MERFISH imaging data were processed with the MERlin^90^ pipeline to decode the spatial location of the mRNA probes. The cells were segmented using Baysor 0.6.2 (ref. 36) with the scale parameter set to 6.5 μm and the minimum molecules per cell parameter set to 50. The decoded molecules were then assigned to the cell boundaries from Baysor to produce a cell-by-gene matrix that lists the number of each molecules decoded for every gene within each cell. Each cell in the matrices was then filtered based on quality control cutoffs of minimum 20 transcripts per cell and minimum five unique genes per cell. The expression matrix for each sample was then concatenated and normalized and log transformed using Scanpy^91^ prior to integration with Harmony^92^. The samples were then subjected to label transfer from the Allen Brain Cell Atlas^49^, following the Seurat integration and label transfer pipeline^30^, and from an atlas of the adolescent mouse brain (http://mousebrain.org/adolescent/downloads.html) with the scVI and scANVI protocol^93^.

#### IBA1 initial segmentations.

The microglial IBA1 stain was imaged across 6–7 imaging planes, spaced by 1.5 μm each, using an anti-rabbit oligonucleotide-conjugated antibody. Python’s scikit-image library was used to create a maximum projection image for each imaging stack. After maximum projection, the images were preprocessed for segmentation after histogram equalization by being converted down to 8-bit images. Then, using OpenCV, background subtraction and edge detection were applied to the 8-bit images to identify the borders of the microglia and remove any imaging noise caused by tissue autofluorescence. Finally, a Gaussian blur and an Otsu segmentation were applied to the images to generate coarse-outlined microglia. This binary image was then converted into a label image such that every segmented IBA1 stain was assigned an identity for downstream processing.

#### Matching transcriptomically derived boundaries to cell masks.

Each brain has a Baysor-derived cell segmentation associated with it; using these geometries, we then align our labeled IBA1 image to cell segmentations. This allowed us to generate a list of cell boundaries that were derived transcriptomically and overlapped with the image analysis-derived cell boundaries. Then, using cell annotations that were generated from the Seurat-based label transfer with the Allen Brain Cell Atlas and cross-referenced against cells labeled using scVI and scANVI, we identified microglial-labeled cell boundaries and binary image labels that were uniquely mapped to one another. The coordinates of these uniquely mapped microglia were then saved for further analysis.

#### High-dimensional embedding of microglial morphology.

Using the microglial coordinates and coarse-grained morphology masks for each cell, a bounding box was generated around every microglia in their raw maximum-projected image. Then, following previously established methodologies for high-dimensional image embeddings^39^, we resized our microglia bounding boxes to 112 × 112 pixels and applied padding with a middle gray color to achieve a 224 × 224 image for each cell. These resized images were then passed through the VGG-19 neural network with pretrained weights from the ImageNet dataset, as accessed through the PyTorch Keras library. After passing the resized images through the neural network, we generated a maximum-pooled vector of the fifth activation layer to create a 512-dimension vector to represent the morphological space of the microglia. The UMAP visualization and clustering, which was used to represent the morphology space, was generated by performing principal component analysis on the 512-dimension vector. To generate the UMAP, a nearest neighbor mapping of the top 10 principal components, capturing approximately 75% of the cumulative explained variance, was used for its calculation. The clustering that was used to represent the morphology space was calculated using the Python kneed library and *k*-means clustering. The kneed library’s KneeLocator function was used to identify the elbow of the *k*-means cluster on an inertia plot. Setting the number of clusters to the identified elbow, we generated a clustering that captured the minimum amount of variance necessary to explain the data.

#### Remapping transcriptomic data to newly segmented microglia.

Wanting to create a more fine-grained representation of the microglial transcriptome than what the Baysor segmentations offered, we used the microglial coordinates to remap transcripts that were directly correlated with the microglial processes. To perform this analysis, we identified the IBA1 stain that was at the center of the saved microglial coordinates and performed an additional round of segmentation on the raw maximum-projected microglia image and DAPI image. Using OpenCV frameworks for adaptive thresholding, with an approximately 200-pixel window size, we generated separate single-cell masks for the DAPI channel and the IBA1 channel aligned to each microglial coordinate. We then used the differences between the DAPI channel and the IBA1 channel to generate a ‘soma’ image mask and a ‘processes’ image mask, with the unity between the two channels being our total cell mask. Then, for each of the individual *z*-planes, we generated a mask of the DAPI, IBA1 and GFAP channels using adaptive thresholding each with a reduced window size. We then, on each individual *z*-plane, took the difference between the reduced window IBA1 processes segmentation and the GFAP segmentation to remove any potential astrocyte false-positive transcripts in the microglial processes. After the removal of potential astrocyte overlap areas, we then found the overlap between the decoded transcript location outputs at each *z*-plane from MERFISH imaging runs and correlated them to our soma, processes and total imaging masks. This process was repeated for all imaging planes to effectively create a three-dimensional reconstruction of the cell and its co-localized transcripts. These separate correlations were saved for each cell, rendering a soma, non-soma and total count vector for each.

#### Annotation of transcriptomically defined microglial clusters.

The total count for each microglia was normalized by dividing each gene’s total transcript count by a cell’s total amount of transcripts and multiplying by 10,000 and then ultimately log transforming the data. The transcriptomic UMAP visualization was created by scaling the log-transformed counts and calculating principal components on all the genes in the dataset. The top 40 principal components were used to generate the nearest neighbor graph for UMAP calculation, and Leiden clustering of the principal components was used to subcluster the microglia. Upon removing subclusters with an increased normalized expression of macrophage markers and individual cells, which lack expression of *Tmem119*, *P2ry12*, *Hexb*, *Mertk*, *Inpp5d* or *Plcg2*, individual subclusters were tested for marker gene expression based on the Wilcoxon rank-sum test to aid in the identification of subcluster identity. Subclustering identification was ultimately concluded based on manual annotation from the microglial subtype marker gene expression, normalized between the different Leiden clusters.

#### Microglial morphological measurements.

Using the same resegmentation approach applied earlier, we wanted to generate detailed morphological measurements of the microglial ramifications and features that were described previously^42–45^. The individual characteristics were calculated by generating a region property for each microglial segmentation and using native scikit-image libraries to calculate the features. In total, pixelwise morphological measurements were calculated for cell area and perimeter, the convex hull area and perimeter, solidity, convexity, roughness, circularity, the convex hull’s span ratio, convex hull circularity, eccentricity, the Euler number, the extent, soma area, soma perimeter, soma circularity, soma-to-cell size ratio and the cell’s ferret diameter. The next set of measurements was a direct result of skeletonization of the microglial segmentation in which we analyzed the total skeleton length in pixels, the mean length of each branch, the total number of branches, the total number of branching points and the number of terminal points; we then calculated Euclidean and path distance of the skeleton branches. Next, we assessed Sholl parameters by calculating the number of intersections between the skeleton branches with circles of varying radii centered around the central node. The Sholl parameters calculated were the ramification index, which is a ratio between the radius of the circle with the greatest number of intersections and the radius of the first non-zero circle, the radius with the greatest number of intersections and the largest radius that had an intersection with the skeleton. Fractal dimension^43^, lacunarity^43^ and tortuosity^94^ were also calculated for the individual microglia. Finally, we assessed the normalized intensity of the IBA1 stain for each of the region properties assessed.

#### Comparison between DEGs from transcriptomically and morphologically derived microglia.

To compare microglial labels that were derived transcriptomically against those that were derived morphologically, we performed differential expression analysis, which was estimated using the Mann–Whitney–Wilcoxon test. We compared the DAM-like microglia to the homeostatic microglia, and then we compared the C1 morphology cluster to the C5 morphology cluster. We categorized a DEG as one that displayed a log_2_ fold change greater than 1 and an adjusted *P* value of less than 0.05. After the identification of DEGs, we identified enriched Gene Ontology Biological Process terms for each set of genes, morphology or transcriptomic-derived clusters, using the Python gseapy package. To compare the glutamate transporter score between C1 and C5 microglia in the homeostatic cells, we scored C1 and C5 cells for log-normalized expression of *Slc1a2*, *Gria2*, *Dlgap2*, *Dlg2* and *Dagla*^47,95–99^ using scanpy’s tl.score_genes functionality and performed a Mann–Whitney–Wilcoxon test between the two distributions to establish statistical significance.

### Identifying ubiquitously expressed and microglia-specific gene sets

To assess cell type specificity of genes in our MERFISH panel, we used 10x v3 data from the Allen Brain Cell Atlas. We subset the data to include only the 500 genes from our MERFISH panel and computed mean log-normalized expression values for both individual classes and subclasses. Cell type specificity was quantified using the tau statistic^100^, calculated from these mean expression values to generate specificity scores ranging from approximately 0.1 to 1.0, where lower values indicate ubiquitous expression and higher values correspond to cell-type-specific expression.

For gene classification, we applied a tau threshold of 0.85 to define cell-type-specific genes^101^. Genes meeting the following criteria were retained for downstream analyses: (1) subclass-level tau ≥ 0.85 with highest mean expression in microglia or (2) tau < 0.80 in both class-level and subclass-level calculations, indicating ubiquitous expression. This filtering approach yielded a final set of 216 genes comprising both ubiquitous and microglia-specific transcripts.

#### Correlation between morphological measurements and transcript measurements.

We used the MERFISH and morphological data for each individual microglia in this analysis. After log normalization of the total transcript counts for each cell, we then performed a training/test split with 75% of the cells in our object being used for training and 25% of the cells being used for subsequent clustering analysis. Using the cells from our training set, we calculated the Spearman correlation coefficient for each morphology–gene pair. Generating a list of the genes that were among the top five correlating for each individual morphological feature, we then clustered the microglial morphological clusters in our test set using the top five correlating genes. This analysis was then repeated on each individual brain region across the mice. Wanting to better understand the correlation between the genes and the features that correlate with higher degrees of ramification, we selected the genes with the highest degree of correlation to the following features: ‘Cell Area’, ‘Convex Hull Perimeter’, ‘Convex Hull Area’, ‘Cell Perimeter’, ‘Number of Branches’, ‘Skeleton Length’, ‘Cell Roughness’, ‘Number of Terminal Points’, ‘Number of Branching Points’, ‘Soma Ratio’, ‘Path Distance’, ‘Fractal Dimension’, ‘Euclidean Distance’, ‘Tortuosity’, ‘Branching Index’ and ‘Dendritic Maximum’. The genes selected were generated from observation of the cluster map created from displaying correlations among the top five correlating genes for each morphology feature; these genes included *Slc1a2*, *Gria2*, *Pink1*, *Ank2*, *Gria1*, *Slc6a11*, *Aldoc*, *Gnas*, *Mfn2*, *Atp2a2*, *Mobp*, *Pcdh9*, *Gfap*, *Aqp4*, *Sdc3*, *Cisd1*, *Sst*, *Spp1*, *Apoe*, *Axl*, *Pcdhb8*, *Cxcl3*, *Rbm3*, *Cemip2*, *Cirbp* and *P2ry12*. These gene and morphology sets were then calculated for the average Spearman correlation in each of the individual regions, where the average was for each gene across correlations for all morphologies. This was used to generate a count for how many regions each gene was a top five correlating gene for.

### Intracellular clustering of transcripts

We first calculated a normalized distance for every transcript from the center of the soma in our C4 and C5 microglia. To do so, for every C4 and C5 microglia in the dataset, we visualized the soma, using a DAPI stain overlayed with the microglia stain, and used the regionprops.centroid function in scikit-image to establish a soma center. We then calculated Euclidean distance from the outermost edge of the microglia and every transcript to the centroid. We then normalized the transcript–centroid distances by the outermost edge–centroid distance for each microglia. These results were then visualized as a cumulative density function for each transcript species.

The degree of clustering was calculated from an adaptation of the DypFISH Ripleyʼs *K* generalization^67^. In brief, we estimated a normalized Ripleyʼs *K* function in three dimensions for each transcript under a homogenous Poisson process to create a Ripleyʼs *H* function for each cellular compartment. We then compared the observed Ripleyʼs *H* function to either a soma-constrained or process-constrained Ripleyʼs *H* function derived from a random distribution of transcripts across the whole cell. Spatial clustering was considered significant at a radius *r* if the computed Ripleyʼs *K* was above the 95% confidence interval or below the 5% confidence interval for the random distribution. We performed this analysis in each of the two compartments individually; the soma clustering was performed only on the transcripts that overlapped with the underlying DAPI stain. The processes degree of clustering was approximated by calculating the degree of clustering for a gene within each of the individual processes and averaging their values together. We then compared the significantly clustered gene’s degree of clustering values at each aging timepoint using the Wilcoxon rank-sum test for genes that were significantly clustered at each age and were either microglial specific or ubiquitously expressed.

### Comparison of soma and process segmented gene co-localization

For each C4 and C5 microglia in the dataset, we calculated the three-dimensional pairwise distance between every transcript within the soma. Then, by using the numpy.percentile function across all of the calculated distances, we found the fifth percentile of 1 μm to be the co-localization radius. We used the subcellular location of each transcript to identify gene–gene pairs that were within 1 μm of each other. We also generated a null distribution of transcript co-localization by randomizing the position of the transcripts within each cell and counting the gene–gene pairs separated by less than 1 μm. Similar to previous methods used for cell–cell interaction^30^, we randomized and repeated this process 1,000 times to generate a null distribution for each cell in the dataset. We then compared the observed number of gene–gene contacts to the null distribution and performed a *z*-test with Benjamini–Hochberg correction. We assumed normality for our null distribution, although it was never formally tested. We aggregated observations across identical gene–gene pairs averaging counts and statistical measures across different morphologies and batches. We then calculated the threshold for significant co-localized counts by calculating the 90th percentile of non-zero gene–gene contacts across our average null distributions for the soma of each cell. From this analysis, we considered a gene–gene pair to be co-localized if it had four or more observations, an adjusted *P* value of less than 0.05 and formed a cluster of more than two genes.

After establishment of gene–gene pairs, we subset our dataset down to look just at the C4 and C5 microglia and genes that were specific to microglia or ubiquitously expressed. We generated networks of genes using Python’s networkx library, with the edges being weighted by the *z*-score between the observed and the null distribution. We then applied scikit-learn’s GaussianMixture, with manually determined cluster numbers for each connected component to separate gene interaction networks into functional clusters. The genes comprising these clusters were then subjected to Gene Ontology analysis for Biological Processes.

#### Comparison of soma-enriched versus process-enriched gene expression and correlation.

To compare the enrichment of certain genes in the different intracellular compartments, we performed differential expression with the Mann–Whitney–Wilcoxon test. This differential expression was performed on only the most ramified microglial set, those microglia mapping to ‘C4’ and ‘C5’ morphologies, so that we maximized the process area in comparison to the soma. Then, independently taking the soma and non-soma counts and log normalizing the two sets as described previously for the same set of cells, we performed the Mann–Whitney–Wilcoxon test between them. We described process-enriched genes as those that held a log_2_ fold change of greater than 1 with an adjusted *P* value of less than 0.05 when compared to the soma-enriched genes, and the same criteria were applied to the soma-enriched genes. This analysis was repeated for the whole dataset, regardless of age, and for the dataset split by age. After the selection of process-enriched and soma-enriched genes for each age in the dataset, we took the genes that were the intersection of the old and young process-enriched and soma-enriched genes. This yielded one set of process-enriched genes and one set of soma-enriched genes. We then calculated the Spearman correlation coefficient between each of the individual genes and every morphological characteristic, regardless of age. We then averaged coefficients for each morphology characteristic for each set of enriched genes. This analysis was then repeated for the process-enriched genes at each age.

### Random forest classifier creation and performance analysis

To assess the predictive capacity of the process-enriched genes as a whole and at each age, we wanted to establish a classifier for the different morphology classes. To build this classifier, we selected the universal process-enriched genes as our features. Then, we categorized morphologies C1 and C2 together as amoeboid, and we categorized C4 and C5 together as ramified and generated a model to predict if the cells were amoeboid or ramified in general and for each age.

To train the random forest models, using scikit-learn’s train_test_split function, we generated an 80/20 training/test split for the microglia both as a whole and at each age. Then, for training, we used scikit-learn’s GridSearchCV to tune the hyperparameters of the random forest classifiers to achieve greatest accuracy for each. Assessing the performance of these models, we used the AUROC, calculated through scikit-learn’s roc_auc_score. Using the mean decrease in impurity calculation for each of the features that were used to train the classifier, we assigned feature importances to each gene as a whole and for the individual ages.

### Immunofluoresence staining and analysis of AXL-positive microglia

Perfused brains from six 24-month-old mice were fixed in 4% paraformaldehyde overnight at 4 °C. After the fixation, brains were submerged in 30% sucrose overnight at 4 °C and then embedded in OCT compound and stored at −80 °C until further use. Brains were sectioned into 40-μm free-floating sections using a cryostat operating at −20 °C. Sections from three male and three female mice were washed with PBS and then blocked and permeabilized with a 2% donkey serum and 0.3% Triton X-100 solution for 1 hour at 4 °C. After incubation, the samples were incubated on a shaker at 4 °C overnight in 1:1,000 dilutions of IBA1 and AXL (Bio-Techne, AF854) antibodies in the blocking solution. After incubation and washing in Tris-buffered saline with Tween 20 (TBST), the samples were then incubated on a shaker at 4 °C overnight in 1:1,000 dilutions of the appropriate species-specific Alexa Fluor secondary antibodies in the blocking solution. Sections were then washed three times in TBST and counterstained with DAPI, at which point they were washed in PBS and mounted on slides using Fluoromount-G Mounting Medium (Invitrogen, 00–4958-02).

Images were captured in the MPM region of the mice on a Leica Stellaris 8 confocal microscope at ×60 magnification with an oil immersion lens, and image analysis was performed in Python. Results were obtained from 3–4 images per mouse across 30-μm *z*-stacks. Each plane in the IBA1 and AXL channels was subjected to normalization, contrast limited adaptive histogram equilization and denoising using OpenCV. The AXL channel was then subjected to an Otsu segmentation on each of the individual frames to identify AXL aggregates with more than 50 pixels (approximately 3 μm^2^). The IBA1 channel was subjected to two layers of segmentation. First, a maximum projection was generated for the IBA1 channel and adaptive thresholding was applied, as done previously, to identify a rough outline of the cell; then, on every plane of the image, adaptive thresholding was applied for the AXL channel, and only those segmentations that intersect the full maximum projection were considered for downstream analysis. After the segmentation, we identified AXL aggregates in microglial processes by identifying overlap between the segmentations. These cells, which were positive for AXL aggregates, were then subjected to cell solidity and fractal dimension analyses, as discussed previously. To assess ramified and amoeboid cells, we set thresholds for both cell solidity and fractal dimension at the median value of the C4 cluster, where values above the threshold for fractal dimension and below the threshold for cell solidity were considered to be ramified and below were considered to be amoeboid. After the analysis, both the single-plane images and three-dimensional renderings were created using ImageJ software (NIH).

### smFISH split probe design and paired immunofluoresence imaging

To verify the co-localization of *Slc1a2* and *Gria2* inside of microglia, we performed smFISH using HCR v3.0 (ref. 102) across 3-month-old and 24-month old mice. In brief, we first designed the probes using National Center for Biotechnology Information Primer-BLAST, which designs internal hybridization oligos and checks for binding specificity through BLASTn. We designed 21-bp primer pairs for an amplicon length of 42 bp with a primer melting temperature between 57 °C and 63 °C and also with a primer GC content between 35% and 70%. Then, 9–10 sets of reverse-complemented forward and reverse primers were concatenated to a flanking sequence for hybridization chain reaction (HCR) and ordered from Integrated DNA Technologies for each gene target and mixed together to form a final stock solution of 10 μM for each target. The exact sequences of reverse-complemented forward and reverse primers are provided in Supplementary Table 4. Brains were sectioned into 30–40-μm sections using a cryostat operating at −20 °C. Sections were then incubated in 4% paraformaldehyde for 12 minutes followed by washing with 1× PBS. After washing of samples, the sections were placed in 70% ethanol and photobleached for 4 hours prior to permeabilization overnight at 4 °C. Samples were then treated with a 5 μg ml^−1^ Proteinase K treatment for 2 minutes at room temperature, at which point they were washed with PBS. Hybridization Buffer was prepared with 2× SSC, 5× Denhardtʼs solution, 10% ethylene carbonate, 10% dextran sulfate, 0.01% SDS and 1 μM probe pool mix for the hybridization reaction. Then, 35 μl of Hybridization Buffer was added to the tissue samples and incubated for 24 hours at 37 °C. After hybridization, the samples were then washed in hybridization wash buffer (0.215 M NaCl, 0.02 M Tris HCl (pH 7.5) and 0.005 M EDTA) for 20–30 minutes at 48 °C. The gene targets were each labeled through HCR v3.0 with hairpin pairs and labeled with Alexa Fluor 647 (*Slc1a2*) and Alexa Fluor 546 (*Gria2*). Amplification Buffer was prepared with 2× SSC, 5× Denhardtʼs solution, 10% dextran sulfate, 0.01% SDS and 0.06 μM HCR hairpins for the amplification reaction. Two microliters of each fluorophore-labeled hairpin at 3 μM corresponding to the target genes was mixed, incubated at 95 °C for 1.5 minutes, covered in aluminum foil and left to cool down at room temperature for 30 minutes to form hairpins before adding it to Amplification Buffer. This amplifixation buffer was then added (50 μl per sample) to the samples and incubated overnight at room temperature. The samples were then washed three times at room temperature with wash buffer.

After washing, the samples were then subjected to either IBA1 or IBA1 and ALDH1L1 staining. In brief, the samples were washed with 1× PBS and covered with Blocking Buffer C Premix (Vizgen, 20300100) and RNase inhibitor (NEB, M0314L) for 1 hour. The samples were then placed in a primary antibody staining solution consisting of Blocking Buffer C Premix, 10% RNase inhibitor and either the IBA1 antibody (Abcam, ab178846) or the IBA1 antibody and an ALDH1L1 antibody (Sigma-Aldrich, MABN495) at a volume dilution of 1:1,000 overnight at 4 °C. Primary antibody incubation was then followed by three washes with 1× PBS. After washing, the samples were then placed in a secondary antibody staining solution consisting of Blocker Buffer C Premix, 10% RNase inhibitor and an anti-rabbit Alexa Fluor-conjugated secondary antibody (Thermo Fisher Scientific, A-11008) or both an anti-rabbit and an anti-mouse Alexa Fluor-conjugated secondary antibody (Thermo Fisher Scientific, A-11004) at a 1:500 dilution for 1 hour. The tissues were then quenched for autofluoresence using TrueBlack (Cell Signaling Technology, 92401S). The tissues were then counterstained with DAPI and mounted onto slides using ProLong Gold Antifade Mountant (Thermo Fisher Scientific, P36930).

The tissue was then imaged on a Leica Stellaris 8 confocal microscope. Next, smFISH images were processed and thresholded on ImageJ software. Representative images of microglia with varying levels of ramification were shown as merged three-dimensional renderings across the 488 (IBA1), 546 (*Gria2*) and 647 (*Slc1a2*) channels. Representative three-dimensional renderings were generated using a consistent workflow: the 488 (IBA1) channel was strictly thresholded across all *z*-planes, and resolved puncta that failed to co-localize with the IBA1 segmentation were removed, leaving only those that co-localized to the IBA1 stain in each plane. The processed images were then displayed using the ImageJ ‘3D Project’ function and rotated to show all orthogonal planes. Additional single-plane orthogonal images were generated for each cell using the ‘Reslice’ function in ImageJ. The positioning of the orthogonal viewing planes was determined by calculating their corresponding distance through the orthogonal *z*-stack.

Representative images of microglia with varying levels of ramification were shown as merged three-dimensional renderings across the 488 (IBA1), 546 (*Gria2*) and 647 (*Slc1a2*) channels, with the original IBA1 *z*-stack displayed alongside background-reduced *Slc1a2* and *Gria2* puncta. Additionally, representative images of microglia from both young and aged brains were shown as merged three-dimensional renderings across the 488 (IBA1), 568 (ALDH1L1) and 647 (*Slc1a2*) channels. For these age-comparison images, the original IBA1 and ALDH1L1 *z*-stacks were displayed with IBA1-localized *Slc1a2* to demonstrate the existence of IBA1-localized *Slc1a2* that does not overlap with ALDH1L1.

### Microglia morphological characteristics across maximum projection depths

To assess whether 10–20-μm tissue sections adequately capture microglial morphology, we analyzed full-thickness IBA1-stained sections from both smFISH and AXL immunohistochemistry experiments. Images were maximum projected across their entire depth, and microglia were segmented using adaptive thresholding (OpenCV). For each segmented cell, we calculated the fractal dimension from the full-volume projection and identified the *z*-plane containing the soma center based on peak IBA1 intensity.

To simulate the effect of thinner sections, we generated maximum projections of varying thicknesses (10 μm, 15 μm and 20 μm) centered on each cell’s soma. For 10-μm projections, we created a series of overlapping slices—starting with the 10-μm slice ending at the soma center and then systematically shifting until the slice began at the soma center. Fractal dimensions were calculated for each projection and averaged per cell. This process was repeated for 15-μm and 20-μm thicknesses.

The relationship between full-thickness and section-limited fractal dimensions was quantified by linear regression (SciPy linregress), with correlation coefficients calculated using sklearn.metrics.

### Statistics and reproducibility

Mann–Whitney–Wilcoxon tests were performed between individual samples, and the resulting *P* values were corrected for multiple hypothesis testing, where applicable, using the Benjamini–Hochberg correction to obtain FDR-adjusted *P* values. The Kruskal–Wallis test was used for comparisons of more than two groups.

No statistical methods were used to predetermine sample sizes, but our sample sizes are similar to those reported previously^31^. No data were excluded from the analyses. Data collection and analysis were not performed blinded to the conditions of the experiments.

## Extended Data

## Supplementary Material

Supplementary protocols and Supplementary Figs. 1−4.

table 4

table 3

table 2

table 1

**Supplementary information** The online version contains supplementary material available at https://doi.org/10.1038/s43587-026-01089-z.

## Data Availability

Decoded MERFISH data, Baysor processed cell boundaries collected during this work and the final annotated objects for all brains are available on figshare (https://figshare.com/articles/dataset/Aging_MERFISH_Brains/27919227). The mouse brain dataset used for scANVI label transfer can be found as ‘l5_all.loom’ at http://mousebrain.org/adolescent/downloads.html. Additionally, the integration with the Allen Brain Cell Atlas was performed on the 10x v3 data from the 20230521 release found here: https://data.nemoarchive.org/other/grant/aibs_internal/zeng/transcriptome/scell/10x_v3/mouse/processed/counts/. The images used to decode cell position and visualize microglia are available upon reasonable request.

## Code availability

All computational analyses were performed using Python 3.9, and we performed cell segmentation using Baysor 0.6.2. All code for the work and this paper are available on GitHub at https://github.com/doughenze/Microglial_Morphology. The environments used to run the analysis are stored as singularity containers are available via Zenodo at https://zenodo.org/records/14611122 (ref. 103).
