# Supplementary material for: Simultaneous spatial transcriptomics and morphology profiling as tools to explore how microglia change with age
Source: Nat Aging. 2026 Mar 10;6(4):869–85. doi: 10.1038/s43587-026-01089-z (PMC13051481; doi:10.1038/s43587-026-01089-z)
Supplement: Supplementary file 1 — Supplementary protocols and Supplementary Figs. 1−4. [file 43587_2026_1089_MOESM1_ESM.pdf]

nature aging

Supplementary information https://doi.org/10.1038/s43587-026-01089-z

# Simultaneous spatial transcriptomics and morphology profiling as tools to explore how microglia change with age

In the format provided by the authors and unedited

# Supplementary Data Figures

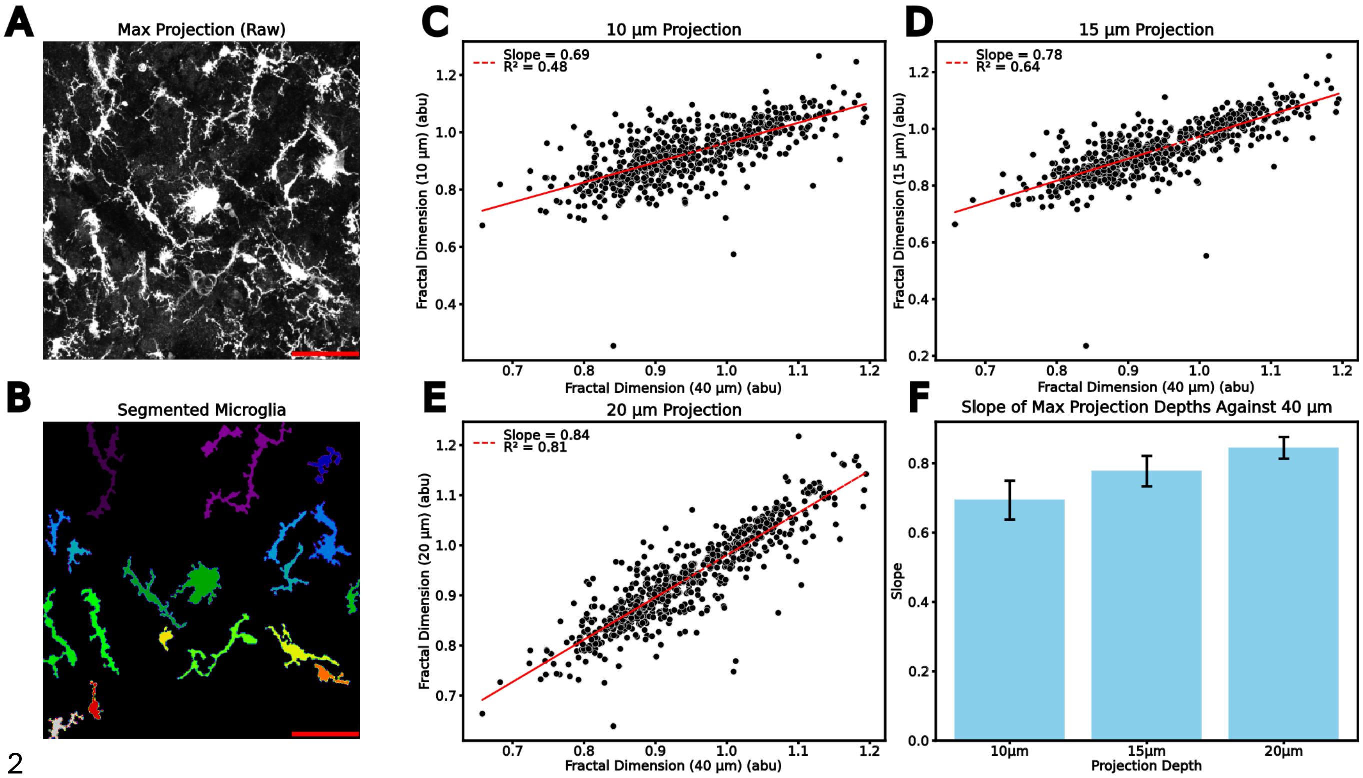


**Supplementary Data Figure 1: Thin section max projections of microglia recapitulate full cell geometry**

**A,** Representative raw image of max-projected microglia across a 40 µm section. **B,** Segmented microglial masks from the same max projected raw image. **C,** Scatter plot displaying the correlation and slope between the full 40-µm max projected fractal dimension and 10-µm, 15-µm (**D**), and 20-µm (**E**) average of max projection fractal dimensions for the same cells. **F,** Bar plot, where the error bars represent the 95% confidence interval of the slope of the lines shown in **C-E** and the bar height corresponds to the slope values in **C-E**, shows the slope between averaged 10-µm, 15-µm, and 20-µm max projection measurements and those of a 40-µm max projection. The measurements were taken over 685 separate microglia which could be resolved across the imaging planes. We assumed normality for calculating our confidence intervals, although it was never formally tested. Scale bars are all 50 µm.

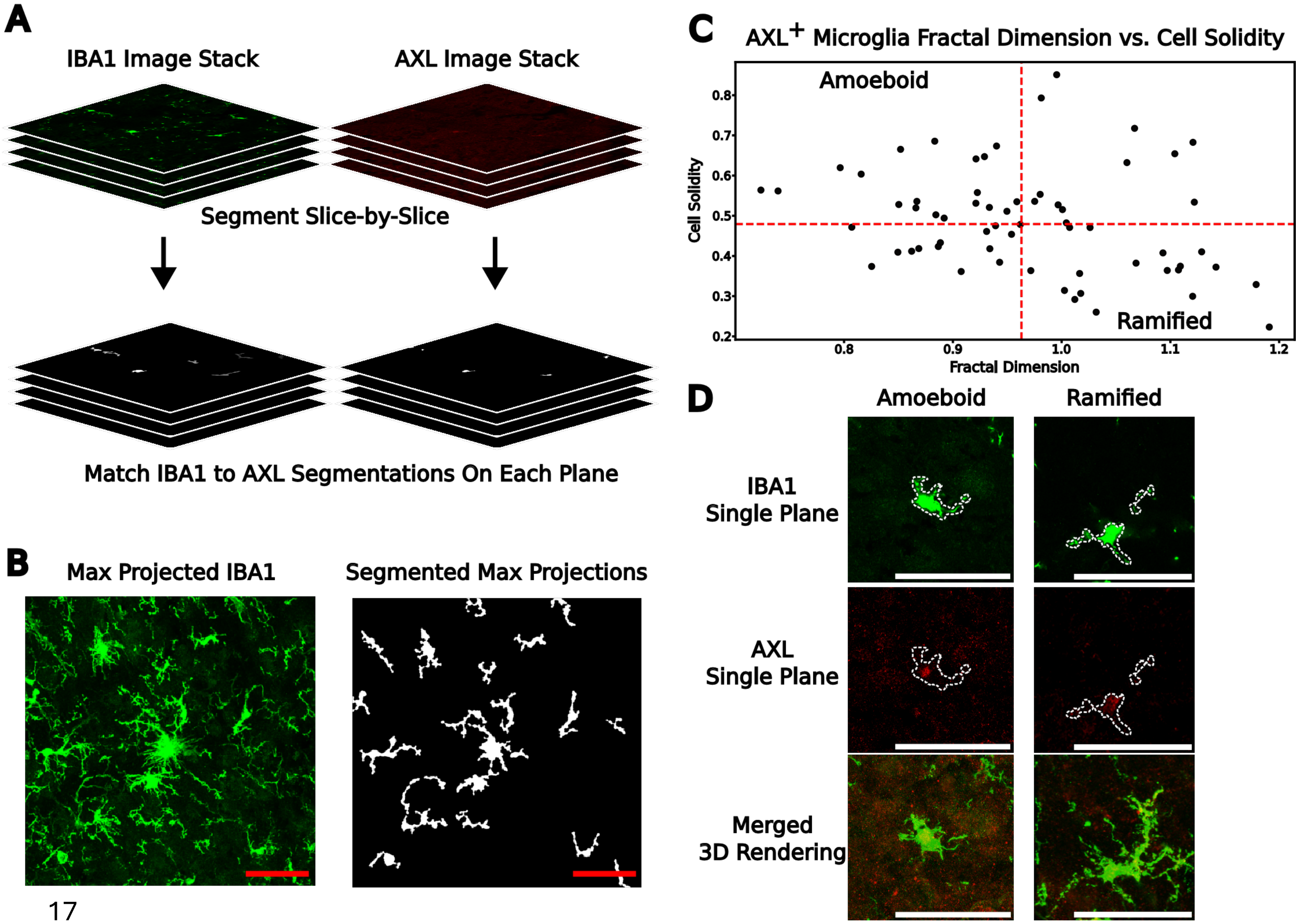


**Supplementary Data Figure 2: AXL+ microglia can be either ramified or amoeboid**
**A,** Strategy to identify AXL+ microglia across 40-µm imaging stacks **B,** Representative max projection (left) and segmentation (right) used to calculate morphological metrics for AXL+ microglia. **C,** Scatter plot of fractal dimension against cell solidity for all AXL+ microglia. The threshold lines (red) are set at the mean value for the C4 cluster to identify the extreme values which can be called ramified (bottom-right quadrant) or amoeboid (top-left quadrant). **D,** Representative single plane AXL and IBA1 images, and a merged 3D rendering of the same amoeboid and ramified cells. Scale bars are all 50 µm.

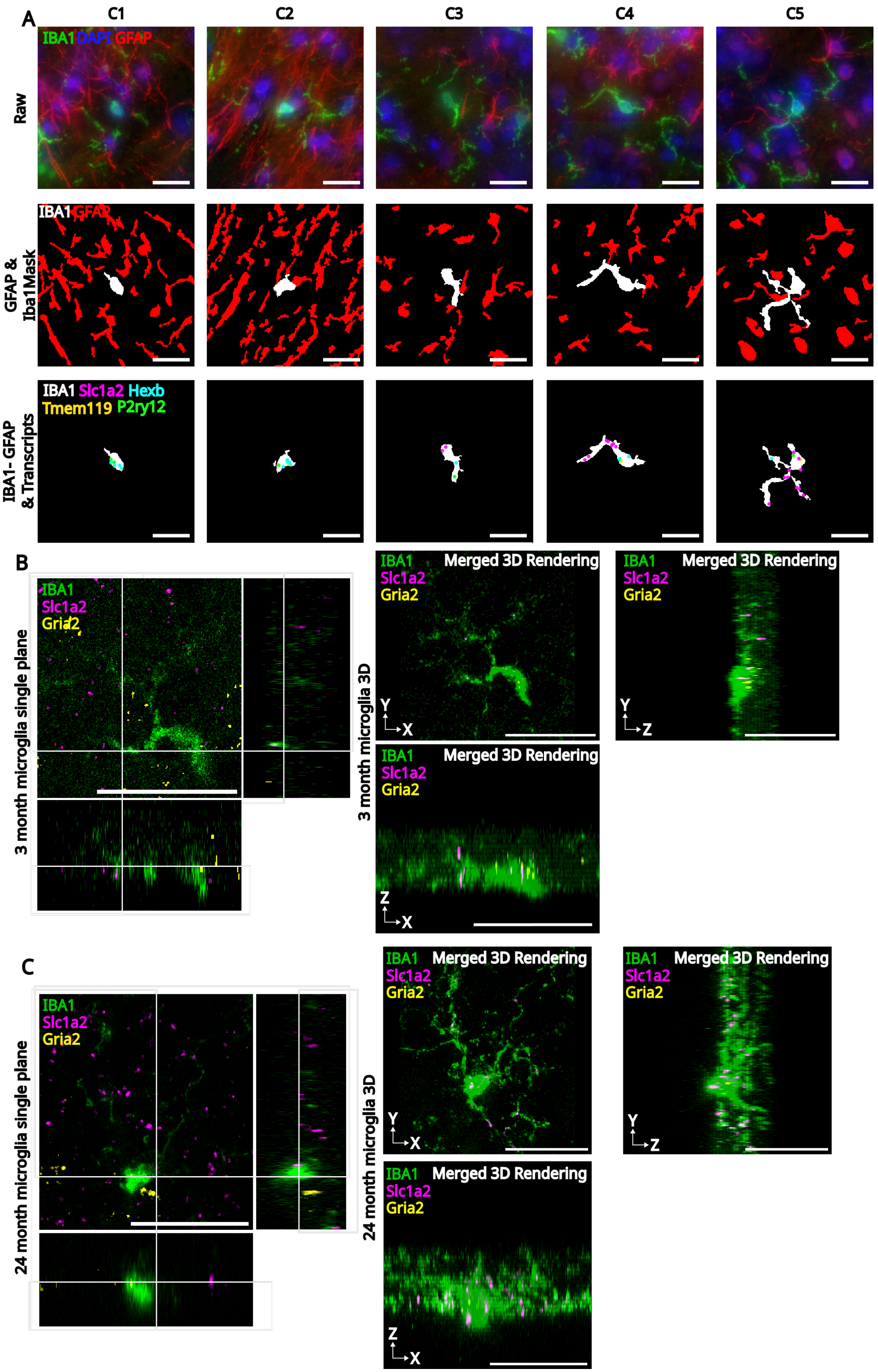

A
C1
C2
C3
C4
C5
Raw
IBA1 DAPI GFAP
GFAP & Iba1Mask
IBA1 GFAP
IBA1- GFAP & Transcripts
IBA1 Slc1a2 Hexb
Tmem119 P2ry12
B
3 month microglia single plane
IBA1
Slc1a2
Gria2
3 month microglia 3D
Merged 3D Rendering
Y
X
Z
C
24 month microglia single plane
24 month microglia 3D

**Supplementary Data Figure 3: Representative Images of MERFISH and smFISH aligned IHC**

**A,** Representative cells from microglial morphological clusters presented as raw images (top), segmented IBA1 and GFAP (middle), and transcript aligned microglia masks (bottom) **B,** (Left) Representative microglia from a young mouse brain, shown as a single plane image with side panels representing 90° rotations of the Z-stacks. (Right) The same cell is also shown as a merged 3D rendering across three orthogonal planes with IBA1 protein staining, and with IBA1 colocalized *Gria2* and *Slc1a2* puncta. **C,** (Left) Representative ramified cell from an aged mouse brain, shown as a single plane image with side panels representing 90° rotations of the Z-stacks. (Right) The same cell is also shown as a merged 3D rendering across three orthogonal planes with IBA1 protein staining, and with IBA1 colocalized *Gria2* and *Slc1a2* puncta. Scale bars are all 25 μm.

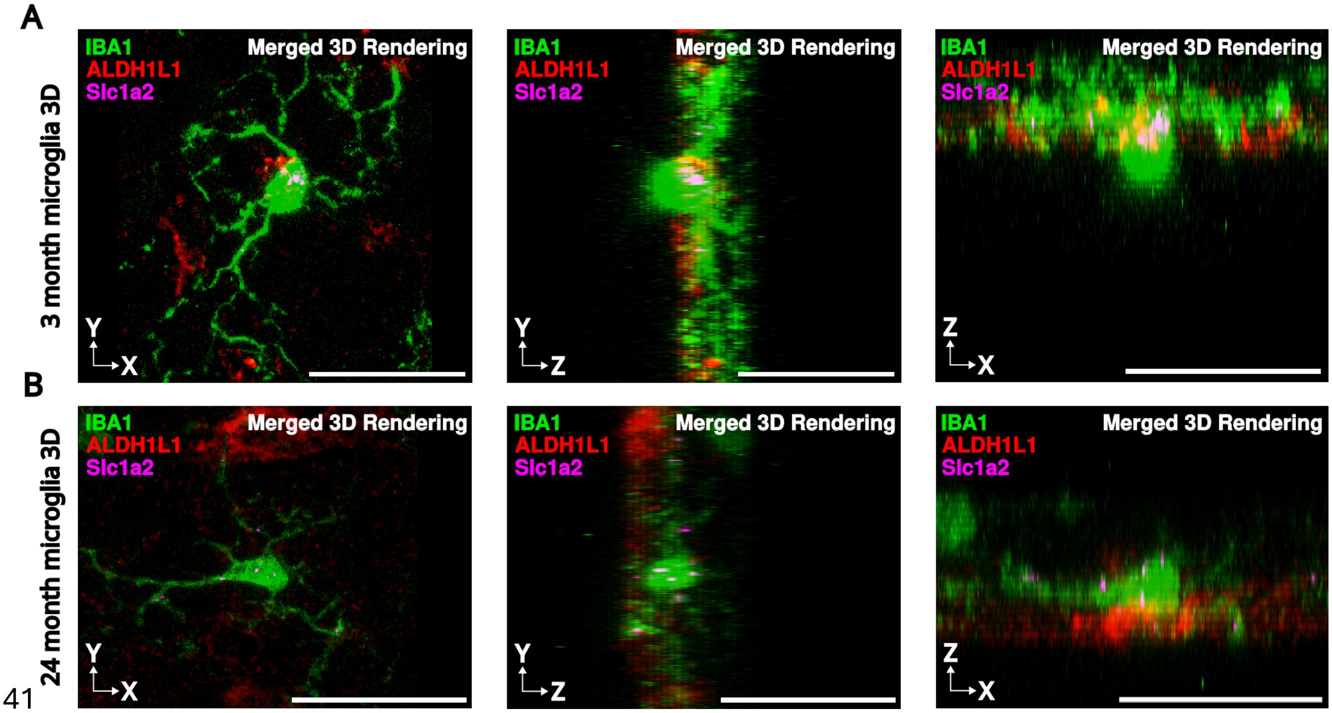


**Supplementary Data Figure 4: Representative Images of smFISH aligned with Microglia and Astrocyte IHC**

**A,** Representative ramified cell from a young mouse brain, shown as a merged 3D rendering across three orthogonal planes with IBA1 protein staining, ALDH1L1 protein staining, and with IBA1 colocalized *Slc1a2* puncta. **B,** Representative amoeboid cell from an old mouse brain, shown as a merged 3D rendering across three orthogonal planes with IBA1 protein staining, ALDH1L1 protein staining, and with IBA1 colocalized *Slc1a2* puncta. Scale bars are all 25 µm.
