# Supplementary material for: Simultaneous spatial transcriptomics and morphology profiling as tools to explore how microglia change with age
Source: Nat Aging. 2026 Mar 10;6(4):869–85. doi: 10.1038/s43587-026-01089-z (PMC13051481; doi:10.1038/s43587-026-01089-z)
Supplement: Supplementary file 2 — Reporting Summary [file 43587_2026_1089_MOESM2_ESM.pdf]

nature portfolio

Corresponding author(s): Dr. Stephen R. Quake

Last updated by author(s): Dec 5, 2025

Reporting Summary

Nature Portfolio wishes to improve the reproducibility of the work that we publish. This form provides structure for consistency and transparency in reporting. For further information on Nature Portfolio policies, see our Editorial Policies and the Editorial Policy Checklist.

Statistics

For all statistical analyses, confirm that the following items are present in the figure legend, table legend, main text, or Methods section.

n/a	Confirmed	
☐	☒	The exact sample size (*n*) for each experimental group/condition, given as a discrete number and unit of measurement
☐	☒	A statement on whether measurements were taken from distinct samples or whether the same sample was measured repeatedly
☐	☒	The statistical test(s) used AND whether they are one- or two-sided<br>*Only common tests should be described solely by name; describe more complex techniques in the Methods section.*
☒	☐	A description of all covariates tested
☐	☒	A description of any assumptions or corrections, such as tests of normality and adjustment for multiple comparisons
☐	☒	A full description of the statistical parameters including central tendency (e.g. means) or other basic estimates (e.g. regression coefficient) AND variation (e.g. standard deviation) or associated estimates of uncertainty (e.g. confidence intervals)
☐	☒	For null hypothesis testing, the test statistic (e.g. *F*, *t*, *r*) with confidence intervals, effect sizes, degrees of freedom and *P* value noted<br>*Give P values as exact values whenever suitable.*
☒	☐	For Bayesian analysis, information on the choice of priors and Markov chain Monte Carlo settings
☒	☐	For hierarchical and complex designs, identification of the appropriate level for tests and full reporting of outcomes
☐	☒	Estimates of effect sizes (e.g. Cohen's *d*, Pearson's *r*), indicating how they were calculated

Our web collection on statistics for biologists contains articles on many of the points above.

Software and code

Policy information about availability of computer code

Data collection	Merscope (Vizgen)
Data analysis	All analyses (except for initial cell segmentation) were performed using Python 3.9.19<br>Transcripts were deconvolved with: MERlin (git: ZhuangLab/MERlin, Apr 20, 2020 version)<br>Initial cell segmentations were generated with Baysor 0.6.2 which was run in julia 1.10.5<br>Morphological embeddings of the samples were generated using tensorflow 2.15.0, opencv-python 4.10.0.84, and protobuf 4.25.3<br>Single cell analysis was performed with Scanpy 1.10.2, harmonypy 0.0.10, and leidenalg 0.10.2<br>Data structures were embedded using anndata 0.10.8<br>Cell type annotation was performed through scvi-tools 0.14.6 and allcools 1.1.0<br>Statistics were performed using scipy 1.13.1<br>Morphology characteristic analysis was performed with scikit-image 0.24.0, scipy 1.13.1, and opencv-python 4.10.0.84<br><br>For reproducibility, all code has been uploaded to a public GitHub repository and all containers containing the necessary environments are held in a public zenodo dataset. Both are linked in the manuscript.

For manuscripts utilizing custom algorithms or software that are central to the research but not yet described in published literature, software must be made available to editors and reviewers. We strongly encourage code deposition in a community repository (e.g. GitHub). See the Nature Portfolio guidelines for submitting code & software for further information.

Data

Policy information about availability of data

All manuscripts must include a data availability statement. This statement should provide the following information, where applicable:

- Accession codes, unique identifiers, or web links for publicly available datasets
- A description of any restrictions on data availability
- For clinical datasets or third party data, please ensure that the statement adheres to our policy

Decoded MERFISH data, Baysor processed cell boundaries collected during this work, and the final annotated object for all brains is available on figshare (https://figshare.com/articles/dataset/Aging_MERFISH_Brains/27919227). The mouse brain dataset used for scANVI label transfer can be found here as l5_all.loom: http://mousebrain.org/adolescent/downloads.html. Additionally the integration with the Allen Brain Cell Atlas was performed on the 20230521 release found here: https://data.nemoarchive.org/other/grant/aibs_internal/zeng/transcriptome/scell/10x_v3/mouse/processed/counts/.

Research involving human participants, their data, or biological material

Policy information about studies with human participants or human data. See also policy information about sex, gender (identity/presentation), and sexual orientation and race, ethnicity and racism.

Reporting on sex and gender	N/A
Reporting on race, ethnicity, or other socially relevant groupings	N/A
Population characteristics	N/A
Recruitment	N/A
Ethics oversight	N/A

Note that full information on the approval of the study protocol must also be provided in the manuscript.

Field-specific reporting

Please select the one below that is the best fit for your research. If you are not sure, read the appropriate sections before making your selection.

☒ Life sciences ☐ Behavioural & social sciences ☐ Ecological, evolutionary & environmental sciences

For a reference copy of the document with all sections, see nature.com/documents/nr-reporting-summary-flat.pdf

Life sciences study design

All studies must disclose on these points even when the disclosure is negative.

Sample size	No statistical methods were used to predetermine the sample sizes. For the MERFISH sequencing experiment 6 mice were sequenced at each age (3 and 24 months) (3 mice for each sex), for a total of 12 mice. Sample size was based on work in a previous publication (PMID: 36580914)
Data exclusions	All data is included.
Replication	All replicates reproduced the phenotypes. Each mouse was imaged entirely separate from the other mice in the study. The number of identified cells and the total number of animals is reported for the experiments.<br>The permutation tests, which consisted of randomly shuffling the decoded transcripts across an individual cells geometry mask were performed 1000 times for each cell independently.
Randomization	Among the 6 animals imaged for each age all 6 were randomly chosen for MERFISH imaging experiments. For each mouse the imaging experiments were definitive, and no randomization was necessary.
Blinding	The investigators were not blinded during experiments and outcome assessment. Blinding during data collection was not needed due to images being taken under the same condition. Quality control filtering was performed blinded to the age of the samples. During the analysis, as the purpose of the study was to identify differences in the transcriptome with age blinding of conditions following quality control filtering was not performed.

Reporting for specific materials, systems and methods

We require information from authors about some types of materials, experimental systems and methods used in many studies. Here, indicate whether each material, system or method listed is relevant to your study. If you are not sure if a list item applies to your research, read the appropriate section before selecting a response.

Materials & experimental systems

n/a	Involved in the study
☐	☒ Antibodies
☒	☐ Eukaryotic cell lines
☒	☐ Palaeontology and archaeology
☐	☒ Animals and other organisms
☒	☐ Clinical data
☒	☐ Dual use research of concern
☒	☐ Plants

Methods

n/a	Involved in the study
☒	☐ ChIP-seq
☒	☐ Flow cytometry
☒	☐ MRI-based neuroimaging

Antibodies

Antibodies used	IBA1 (Abcam; REF ab178846); AXL (Bio-Techne; REF AF854)
Validation	Antibody was validated by the manufacturer.

Animals and other research organisms

Policy information about studies involving animals; ARRIVE guidelines recommended for reporting animal research, and Sex and Gender in Research

Laboratory animals	All experiments used C57BL/6J mice (stock #000664), including both young and aged mice, were purchased from Jackson Laboratories
Wild animals	No wild animals were used in the study
Reporting on sex	There was equal representation of male and female mice at each age.
Field-collected samples	No field collected samples were used in this study.
Ethics oversight	All animal care and experimental procedures complied with the Guide for the Care and Use of Laboratory Animals of the National Institutes of Health and were approved by Stanford University's Administrative Panel on Laboratory Animal Care (APLAC). APLAC #29100

Note that full information on the approval of the study protocol must also be provided in the manuscript.

Plants

Seed stocks	No plants were used in this study.
Novel plant genotypes	No plants were used in this study.
Authentication	No plants were used in this study.
